# The association of *BTLA* gene polymorphisms with non-small lung cancer risk in smokers and never-smokers

**DOI:** 10.3389/fimmu.2022.1006639

**Published:** 2023-01-19

**Authors:** Anna Andrzejczak, Anna Partyka, Andrzej Wiśniewski, Irena Porębska, Konrad Pawełczyk, Kuba Ptaszkowski, Piotr Kuśnierczyk, Monika Jasek, Lidia Karabon

**Affiliations:** ^1^Laboratory of Genetics and Epigenetics of Human Diseases, Department of Experimental Therapy, Hirszfeld Institute of Immunology and Experimental Therapy, Polish Academy of Sciences, Wrocław, Poland; ^2^Laboratory of Immunopathology, Department of Experimental Therapy, Hirszfeld Institute of Immunology and Experimental Therapy, Polish Academy of Sciences, Wrocław, Poland; ^3^Laboratory of Immunogenetics and Tissue Immunology, Department of Clinical Immunology, Hirszfeld Institute of Immunology and Experimental Therapy, Polish Academy of Sciences, Wrocław, Poland; ^4^Department of Pulmonology and Lung Oncology, Wrocław Medical University, Wrocław, Poland; ^5^Departament of Thoracic Surgery, Lower Silesian Centre of Oncology, Pulmonology and Haematology, Wrocław, Poland; ^6^Department of Clinical Biomechanics and Physiotherapy in Motor System Disorders, Wrocław Medical University, Wrocław, Poland

**Keywords:** BTLA, SNP, NSCLC, smokers, never-smokers, disease risk, overall survival

## Abstract

**Introduction:**

Lung cancer is the predominant cause of death among cancer patients and non-small cell lung cancer (NSCLC) is the most common type. Cigarette smoking is the prevailing risk factor for NSCLC, nevertheless, this cancer is also diagnosed in never-smokers. B and T lymphocyte attenuator (BTLA) belongs to immunological checkpoints which are key regulatory molecules of the immune response. A growing body of evidence highlights the important role of BTLA in cancer. In our previous studies, we showed a significant association between *BTLA* gene variants and susceptibility to chronic lymphoblastic leukemia and renal cell carcinoma in the Polish population. The present study aimed to analyze the impact of *BTLA* polymorphic variants on the susceptibility to NSCLC and NSCLC patients’ overall survival (OS).

**Methods:**

Using TaqMan probes we genotyped seven *BTLA* single-nucleotide polymorphisms (SNPs): rs2705511, rs1982809, rs9288952, rs9288953, rs1844089, rs11921669 and rs2633582 with the use of ViiA 7 Real-Time PCR System.

**Results:**

We found that rs1982809 within *BTLA* is associated with NSCLC risk, where carriers of rs1982809G allele (AG+GG genotypes) were more frequent in patients compared to controls. In subgroup analyses, we also noticed that rs1982809G carriers are significantly overrepresented in never-smokers, but not in smokers compared to controls. Additionally, the global distribution of the haplotypes differed between the never-smokers and smokers, where haplotypes A G G C A, C G A C G, and C G A T G were more frequent in never-smoking patients. Furthermore, the presence rs1982809G (AG+GG genotypes) allele as well as the presence of rs9288953T allele (CT+TT genotypes) increased NSCLC risk in females’ patients. After stratification by histological type, we noticed that rs1982809G and rs2705511C carriers were more frequent among adenocarcinoma patients. Moreover, rs1982809G and rs2705511C correlated with the more advanced stages of NSCLC (stage II and III), but not with stage IV. Furthermore, we showed that rs2705511 and rs1982809 significantly modified OS, while rs9288952 tend to be associated with patients’ survival.

**Conclusion:**

Our results indicate that *BTLA* polymorphic variants may be considered low penetrating risk factors for NSCLC especially in never-smokers, and in females, and are associated with OS of NSCLC patients.

## 1 Introduction

In 2020, lung cancer was the most common cause of death among cancer patients (1 796 144 deaths) and the second most frequently diagnosed cancer type on the new cancer cases list (2 206 771 new cases), based on data from 185 countries (1). The mortality rate for lung cancer is very high, more than 80% of patients die from this cancer. Late diagnosis and a high degree of malignancy are the main reasons for such high mortality (2, 3). Smoking is a predominant risk factor for lung cancer. Depending on the world region 60% up to 90% of patients are active smokers on the day of diagnosis or smoked in the past. The survival rate among smoking patients decreases with the increasing value of the pack-years indicator (a unit for measuring the amount a person has smoked over a long period of time, calculated by multiplying the number of packs of cigarettes smoked per day by the number of years the person has smoked) (2, 3). However, smoking is not the only risk factor. Several studies showed that only a small percentage of active smokers will develop lung cancer in the future. What’s more, although the risk of lung cancer for never-smokers is 20-50 times lower than that of smokers (3), nevertheless people who never smoked account for 10-20% of all lung cancer cases among Caucasian and other populations (2, 4). This indicates that apart from smoking also other risk factors including genetic factors exist. Never-smokers are defined as individuals who had never smoked or who had smoked less than 100 cigarettes in their lifetime (2). They are more frequently young, female, and more symptomatic at the time of diagnosis than smokers (5).

Non-small cell lung cancer (NSCLC) is the most common type of lung cancer accounting for 80% to 85% of all lung cancer cases. Histological classification of lung cancer published in 2015 by the World Health Organization (WHO) divides NSCLC into three main histological types: adenocarcinoma (AD), squamous cell carcinoma (SqCC), and large cell carcinoma (LCC) (6). Never-smokers, when getting lung cancer, almost always develop NSCLC and adenocarcinoma is a major histological type of NSCLC in this group (7–9). However, data on lung cancer incidence in never-smokers is still limited firstly because of the lack of information on the smoking status in the patient’s medical history and secondly of a small number of never-smoking patients.

Immunological checkpoints (ICs) are key regulatory molecules highly altered in most types of cancer. ICs are responsible for the regulation of the immune response by maintaining self-tolerance and adjustment of the duration and severity of the immune response. In various cancers tumor cells overexpress ICs on their surface to hide from immune system surveillance (10, 11). Next to widely studied ICs like PD-1 and CTLA-4 recently also B and T lymphocyte attenuator (BTLA) emerged as important IC in cancer (12, 13). BTLA belongs to the immunoglobulin superfamily (Ig SF) and is expressed on the surface of B cells, T cells, natural killer (NK) cells, dendritic cells (DCs), and macrophages (14, 15). BTLA binding to herpes virus entry mediator (HVEM) acts as a negative regulator of TCR/BCR-mediated signaling pathways leading to inhibition of T and B cell activation, proliferation, and proinflammatory cytokines production (16). Recent studies showed that BTLA can also act as a costimulatory ligand for HVEM by activating NF-κB pathway (17). Since BTLA plays a significant role in maintaining self-tolerance, BTLA-HVEM pathway disturbance has been demonstrated to be involved in the pathogenesis of autoimmune diseases (18, 19), infections (20, 21), and cancer (22, 23). Our previous studies showed an association between genetic variations in the gene encoding BTLA and chronic lymphocytic leukemia (CLL) risk (24), abnormal expression of BTLA in CLL cells (22) as well as miR-155-5p regulation of BTLA expression in CLL B cells (25).

**Figure 1:**
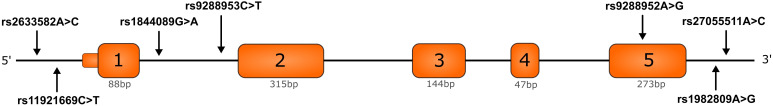
Structure of *BTLA* gene and localization of studied *BTLA* single nucleotide polymorphisms. Orange boxes indicate exons and black lines introns, 5’UTR and 3’UTR regions.

In recent years scientists focused on searching for reliable prediction markers of cancer development. Genetic variations including single nucleotide polymorphisms (SNPs) are considered as cancer potential prediction markers. *BTLA* gene variations have been connected with the risk of cancer development in a few previous studies (26–30) including our recent studies on CLL (24) and renal cell carcinoma (RCC) (31). The association between *BTLA* SNPs and lung cancer susceptibility has been studied previously in Chinese population (rs1982809, rs16859629, rs2171513, rs3112270) (32) and Tunisian population (rs1982809, rs9288953, rs9288952) (33). However, there is no study describing the relationship between *BTLA* SNPs and the risk of NSCLC in the Caucasian population. In the current study, based on our previous findings and literature overview we selected: rs2705511, rs1982809, rs9288952, rs9288953, and rs1844089. Additionally, we selected two new never investigated before *BTLA* SNPs: rs11921669 and rs2633582 located in *BTLA* promoter region (**Figure 1**). This time we aimed to study the association of *BTLA* SNPs with NSCLC risk and overall survival (OS), while we especially focused on this problem in the aspect of cigarette smoking.

## 2 Materials and methods

### 2.1 Control group

Control group depending on studied polymorphism comprised of 474 (266 males and 208 females) (rs2705511, rs1982809, rs9288952, rs9288953, rs1844089) or 309 (180 males and 129 females) (rs11921669, rs2633582) subjects originated from the same geographic region as patients. Differences in the size of the control groups are due to combining genotyped controls from our previous studies (rs2705511, rs1982809, rs9288952, rs9288953, and rs1844089) with newly genotyped controls (rs11921669, rs2633582). Blood samples from healthy subjects were collected by Wrocław blood bank or donated by employees of the Hirszfeld Institute of Immunology and Experimental Therapy. The studies involving human participants were reviewed and approved by the Bioethical Committee of Wrocław Medical University, Wrocław, Poland. The patients/participants provided their written informed consent to participate in this study.

### 2.2 NSCLC patients

The group of patients enrolled in this study is in most cases the same as the group described in Wiśniewski et al. (34), however, the survival data were updated and some missing data concerning histological type were added. Altogether 383 patients (277 males and 106 females) were enrolled by the Department of Pulmonology and Lung Cancer, Wrocław Medical University, Wrocław, and by the Thoracic Surgery Center, Lower Silesian Centre of Lung Diseases, Wrocław. Patients were diagnosed with NSCLC and histological type of lung cancer (adenocarcinoma (AC), squamous cell carcinoma (SqCC), or large cell carcinoma (LCC)) was identified according to the World Health Organization (WHO 2015) classifications. Patient samples were collected for the period from 2005 to 2017. Due to the changes in requirements related to the degree of histological classification associated with the available treatment options some samples were not classified to the level of NSCLC histological subtype. In most cases, the pathologic stage of cancer was also evaluated according to the International System for Staging Lung Cancer (35), as described in (34). NSCLC patients with a history of primary cancer other than lung cancer as well as palliative patients with undetermined disease stage due to general status and/or serious unstable co-morbidities were excluded from the study. Disease progression was determined according to the TNM staging system (36) based on radiological studies and endoscopic techniques. Chest CT, PET-CT, and, if necessary, brain MRI/CT and bone scintigraphy were performed. Bronchofiberoscopy was performed in all patients. If mediastinal lymph node status needed to be verified, additional EBUS-TBNA or mediastinoscopy was performed. When surgery as curative treatment was performed, the final clinical stage was verified by histopathological examination of postoperative specimens, especially lymph nodes. The ECOG scale was used to evaluate the patients’ general status and quality of life. Patients underwent surgery with adjuvant or neoadjuvant chemotherapy (two or three courses at three-week intervals) as needed, radiotherapy, radiochemotherapy, chemotherapy, or palliative treatment only, depending on the stage of the disease, according to local recommendations. Overall survival was assessed from the date of NSCLC diagnosis to death from any cause or up to 31.12.2021 when data collection was completed.

Based on interviews with patients about their smoking history, we divided patients into two groups: never smoking and smoking (active-smoker or past-smoker who quit at least 1 year before diagnosis). In the group of smokers, patients were further divided depending on the number of cigarette packs smoked daily (pack-years). Patients characteristic as well as information about the smoking status of patients is presented in **Table 1**.

**Table 1:** Characteristics of NSCLC group.

Variable		All n=383 (%)	Male n=277 (%)	Female n=106 (%)
Age at diagnosis	**Median**	63	63	63
	**Mean**	63.92	64.21	63.17
	**Q1-Q3**	58-70	59-70	57-69
	**Min, Max**	35, 87	40, 87	35, 86
Smoking history (pack-years)	**0**	50 (13.0)	24 (9.0)	26 (25.2)
	**1-10**	3 (0.7)	1 (0.3)	2 (1.9)
	**11-20**	56 (14.6)	32 (12.0)	24 (23.3)
	**21-30**	87 (22.7)	63 (23.7)	24 (23.3*)*
	**31-40**	95 (24.8)	75 (28.2**)**	20 (29.4)
	**>40**	78 (20.4)	71 (26.7)	7 (6.8)
Histological type	**adenocarcinoma**	111 (29.5)	68 (25.0)	43 (40.9)
	**squamous cell carcinoma**	118 (31.3)	92 (33.8)	26 (24.8)
	**large cell carcinoma**	22 (5.8)	16 (5.9)	6 (5.7)
	**Unknown**	126 (33.4)	96 (35.3)	30 (28.6)
Stage of disease	**I**	85 (22.2)	56 (20.5)	29 (28.2)
	**II**	51 (13.3)	40 (14.7)	11 (10.8)
	**III**	116 (30.3)	83 (30.4)	33 (32.0)
	**IV**	124 (32.4)	94 (34.4)	30 (29.1)
	**Unknown**	7 (1.8)	4 (1.5)	3 (2.9)
Treatment	**Surgery**	174 (45.4)	118 (42.6)	56 (52.8)
	**No surgery**	154 (40.2)	118 (42.6)	36 (34.0)
	**Unknown**	55 (14.4)	41 (14.8)	14 (13.2)

The studies involving human participants were reviewed and approved by the Bioethical Committee of Wrocław Medical University, Wrocław, Poland. The patients/participants provided their written informed consent to participate in this study.

### 2.3 SNPs selection

Five of seven selected *BTLA* SNPs: rs2705511, rs1982809, rs9288952, rs9288953, and rs1844089 have been studied previously by our group in RCC (31) and/or CLL (24), and also by others in breast cancer, colorectal cancer, rectal cancer, esophageal squamous cell carcinoma and esophagogastric junction adenocarcinoma (26–30). Additionally, we selected two new (rs11921669, rs2633582), previously not studied SNPs of *BTLA* 5’UTR region. According to *in silico* analysis rs11921669 and rs2633582 are located in the potential transcription factors binding sites within the promoter region of *BTLA* gene. Extended information about each SNP is provided in **Supplementary Table 1**.

### 2.4 DNA isolation and SNPs genotyping

Genomic DNA was isolated from refrozen blood samples by QIAamp DNA Blood Mini Kit (Qiagen, Germany) according to the manufacturer’s instructions. All SNPs were genotyped using TaqMan Genotyping Master Mix (Applied Biosystems. Foster City, USA) and TaqMan assays: rs2705511 (C:16272823_10), rs1982809 (C:_1175848_20), rs9288952 (C:_1175845_10), rs9288953 (C:_1175838_10), rs1844089 (C:_26921149_20), rs11921669 (C:_1175824_10), and rs2633582 (C:_1175825_10). All reactions were run on the ViiA7 Real-Time PCR system (Applied Biosystems, Singapore).

### 2.5 Statistical analysis

Statistical analysis was performed using the Statistica 13.1 program (TIBCO, Inc., Palo Alto, USA) and PQStat v.1.8.0.476 (PQStat Software, Poznan, Poland). For measurable variables, the means, medians and standard deviations were calculated. All investigated quantitative variables were checked with the Shapiro-Wilk test to establish the type of distribution. For all genotyped *BTLA* polymorphisms the evaluation of Hardy-Weinberg equilibrium (HWE) was performed independently for NSCLC patients and control group by comparing the observed and expected frequencies of genotypes using χ2 test. The χ2 test was used to compare categorical data between NSCLC patients and controls as well as between smoking and never-smoking patients. Odds ratios (OR) and 95% confidence intervals (95% CI) were calculated using the binary logistics regression model to evaluate the relationship between *BTLA* SNPs and susceptibility to NSCLC. The linkage disequilibrium (LD) calculation and haplotype frequencies for pairs of alleles were determined using the SHEsis software (37, 38). Haplotypes with frequencies below 0.01 were not considered. Differences between groups were considered statistically significant if p<0.05.

Survival analysis was performed using the Kaplan-Meier method. The log-rank test was used to compare patient survival against selected clinical variables. The Cox proportional hazards model was used to assess the effect of qualitative or quantitative variables on survival. The analysis included categorical variables and continuous variables. Due to the fact that in the univariate model, the smoking history (pack-years) variable was analyzed as continuous and categorical variables, for the final multivariate model, the smoking history (pack-years) variable was selected depending on the better fit of the model based on the assessment of the goodness of fit (AIC). The results were considered statistically significant if p<0.05. The model building process was performed using a stepwise method and a set of standard measures of goodness of fit (AIC, R2) was used to evaluate the model. The results were considered statistically significant if p <0.05.

## 3 Results

### 3.1 Association between *BTLA* SNPs and susceptibility to NSCLC

Detailed analysis of genotypes and alleles distribution for all selected *BTLA* SNPs in NSCLC patients and controls are presented in **Table 2**. We found that rs1982809 tended to be associated with susceptibility to NSCLC in the overall analysis. Genotypes distribution analysis showed that rs1982809G allele carriers (AG+GG genotypes) were more frequent in the NSCLC group compared to healthy controls (45.29% vs. 38.82%) which indicates that the presence of G allele at rs1982809 SNP increases susceptibility to NSCLC (OR 1.304, CI95% 0.99-1.71, p=0.057). Analysis of allele distribution also confirmed that allele G in rs1982809 significantly increases the risk of NSCLC (OR 1.254, CI95% 1.01-1.57, p=0.046). For the other studied SNPs: rs2705511, rs9288952, rs9288953, rs1844089, rs11921669, and rs2633582 we did not observe any association with susceptibility to NSCLC in the overall analysis.

**Table 2:** Genotypes and alleles distribution of *BTLA* SNPs among NSCLC patients and controls.

			Cases		Controls			
SNP	Genotype	Allele	N	%	N	%	OR (CI95%)	p value
rs2705511	**AA**		208	54.59	274	57.81	1	0.531
	**AC**		143	37.53	170	35.86	1.108 (0.83-1.47)	
	**CC**		30	7.87	30	6.33	1.317 (0.77-2.24)	
	**AC+CC**		173	45.41	200	42.19	1.139 (0.87-1.50)	0.347
	**AA+AC**		351	92.13	444	93.67	0.791 (0.47-1.33)	0.380
		**A**	559	73.36	718	75.74	1	
		**C**	203	26.64	230	24.26	1.134 (0.91-1.41)	0.261
rs1982809	**AA**		209	54.71	290	61.18	1	0.140
	**AG**		146	38.22	159	33.54	1.274 (0.96-1.67)	
	**GG**		27	7.07	25	5.27	1.495 (0.85-2.64)	
	**AG+GG**		173	45.29	184	38.82	1.304 (0.99-1.71)	**0.057**
	**AA+AG**		355	92.93	449	94.73	0.733 (0.42-1.28)	0.275
		**A**	564	73.82	739	77.95	1	
		**G**	200	26.18	209	22.05	1.254 (1.00-1.57)	**0.046**
rs9288952	**AA**		338	88.25	421	88.82	1	0.783
	**AG**		42	10.97	51	10.76	1.028 (0.67-1.58)	
	**GG**		3	0.78	2	0.42	–	
	**AG+GG**		45	11.75	53	11.18	1.059 (0.70-1.61)	0.795
	**AA+AG**		380	99.22	472	99.58	–	
		**A**	718	93.73	893	94.20	1	
		**G**	48	6.27	55	5.80	1.087 (0.73-1.62)	0.687
rs9288953	**CC**		87	36.10	188	40.00	1	0.572
	**CT**		121	50.21	218	46.38	1.198 (0.86-1.67)	
	**TT**		33	13.69	64	13.62	1.119 (0.69-1.82)	
	**CT+TT**		154	63.90	282	60.00	1.178 (0.86-1.62)	0.312
	**CC+CT**		208	86.31	406	86.38	0.998 (0.63-1.55)	0.978
		**C**	295	61.20	594	63.19	1	
		**T**	187	38.80	346	36.81	1.09 (0.87-1.37)	0.464
rs1844089	**GG**		320	85.11	395	83.33	1	0.385
	**AG**		56	14.89	77	16.24	0.900 (0.62-1.31)	
	**AA**		0	0	2	0.42	–	
	**AG+AA**		56	14.89	79	16.67	0.877 (0.61-1.27)	0.483
	**AG+GG**		376	100	472	99.58	–	
		**G**	696	92.55	867	91.46	1	
		**A**	56	7.45	81	8.54	0.86 (0.61-1.23)	0.409
rs11921669	**CC**		362	95.01	298	96.13	1	0.408
	**CT**		17	4.46	12	3.87	1.153 (0.55-2.42)	
	**TT**		2	0.52	0	0.00	–	
	**CT+TT**		19	4.99	12	3.87	1.285 (0.62-2.56)	0.481
	**CC+CT**		379	99.48	310	100.00	–	
		**C**	741	97.24	608	98.06	1	
		**T**	21	2.76	12	1.94	1.511 (0.70-2.86)	0.321
rs2633582	**AA**		322	84.29	265	85.76	1	0.192
	**AC**		56	14.66	44	14.24	1.045 (0.68-1.60)	
	**CC**		4	1.05	0	0.00	–	
	**AC+CC**		60	15.71	44	14.24	1.119 (0.73-1.70)	0.592
	**AA+AC**		378	98.95	309	100.00	–	
		**A**	700	91.62	574	92.88	1	
		**C**	64	8.38	44	7.12	1.189 (0.80-1.77)	0.387

Significant results are bolded.

### 3.2 Linkage disequilibrium and haplotype analysis

The SHEsis online software (38) was used to perform linkage disequilibrium and haplotype analysis. LD analysis showed a significant LD relationship between rs1844089 and rs2633582 (r^2^ = 0.976). No strong LD relationship was observed among other studied *BTLA* SNPs. Three pairs of SNPs were in moderate LD relationship (rs2705511 and rs1982809, r^2^ = 0.588; rs9288952 and rs1844089, r^2^ = 0.576; rs2633582 and rs9288952, r^2^ = 0.572) (**Table 3**).

**Table 3:** Linkage disequilibrium between studied *BTLA* polymorphisms in NSCLC patents and controls.

r^2^	rs1982809	rs9288952	rs9288953	rs1844089	rs11921669	rs2633582
**rs2705511**	0.588	0.045	0.071	0.028	0.007	0.030
**rs1982809**	–	0.140	0.099	0.093	0.004	0.092
**rs9288952**	–	–	0.029	0.576	0.004	0.572
**rs9288953**	–	–	–	0.056	0.019	0.056
**rs1844089**	–	–	–	–	0.327	**0.976**
**rs11921669**	–	–	–	–	–	0.327

Significant results are bolded.

Since two SNPs (rs11921669 and rs2633582) have been genotyped on different group of controls than the rest five SNPs (see Material and Methods) as well as because we did not observe any relationship of rs11921669 and rs2633582 with NSCLC risk we decided to omit these SNPs in further haplotype analysis. Haplotype analysis showed 10 *BTLA* haplotypes with frequency above 0.01. The global distributions of the haplotypes did not differ significantly between NSCLC patients and controls, however we noticed that haplotype C G A T G (rs2705511, rs1982809, rs9288952, rs9288953, rs1844089) was overrepresented in NSCLC patients (OR 1.39, p=0.019) (**Table 4**) and significantly increased the NSCLC risk, while after applying Bonferroni correction this association lost significance.

**Table 4:** *BTLA* haplotypes frequency in NSCLC patients and controls.

Haplotype*	Patients [%]	Controls [%]	Odds Ratio [95%CI]	p value
**A A A C A**	2	2.2	0.931 [0.477~1.819]	0.835
**A A A C G**	46.5	47.9	0.936 [0.771~1.137]	0.506
**A A A T G**	20.4	21.3	0.943 [0.744~1.196]	0.627
**A G A T G**	1.2	1.9	0.650 [0.290~1.455]	0.291
**A G G C A**	1.7	1.5	1.147 [0.537~2.447]	0.723
**C A A C G**	2.7	4.1	0.650 [0.377~1.122]	0.119
**C A A T G**	1.4	1.4	0.998 [0.438~2.277]	0.996
**C G A C G**	2.7	2.5	1.078 [0.590~1.972]	0.807
**C G A T G**	15.9	11.9	1.393 [1.055~1.841]	**0.019**
**C G G C A**	3.4	3.0	1.150 [0.666~1.985]	0.616
**Total**	97.9%	97.7%		
	Global χ2 = 9.03, df=9, p=0.43			

* BTLA haplotype order: rs2705511, rs1982809, rs9288952, rs9288953, rs1844089. Significant results are bolded.

### 3.3 Association of *BTLA* polymorphisms with clinical features of NSCLC patients in subgroup analysis

#### 3.3.1 Analysis of *BTLA* gene variations and NSCLC risk in relation to smoking status

Since we lack full data on smoking status in the control group, we decided to compare smoking and never-smoking patients to overall control. We noticed a significant difference in rs1982809 genotypes frequencies in never-smoking patients (p=0.043) compared to controls. In details, homozygous never-smoking individuals were 3 times more prone to develop NSCLC than patients possessing A allele (AG+AA genotype) (OR 3.03 95%CI 1.27-7.14, p=0.014) **Table 5**.

**Table 5:** Genotypes and alleles frequencies of *BTLA* SNPs and NSCLC risk in Smokers and Never-Smokers.

SNP	Genotype	Allele	Controls		Smokers		Never-Smokers		Smokers vs. Controls		Never-Smokers vs. Controls	
			N	%	N	%	N	%	OR (CI95%)	p value	OR (CI95%)	p value
**rs2705511**	**AA**		274	57.81	175	55.21	25	50.00	1	0.687	1	0.265
	**AC**		170	35.86	118	37.22	19	38.00	1.09 (0.80-1.47)		1.23 (0.66-2.29)	
	**CC**		30	6.33	24	7.57	6	12.00	1.26 (0.71-2.21)		2.29 (0.90-5.87)	
	**AC+CC**		200	42.19	142	44.79	25	50.00	1.11 (0.83-1.48)	0.470	1.37 (0.77-2.44)	0.289
	**AA+AC**		444	93.67	293	92.43	44	88.00	0.82 (0.47-1.43)	0.498	0.47 (0.19-1.16)	0.132
		**A**	718	75.74	468	73.82	69	69.00	1		1	
		**C**	230	24.26	166	26.18	31	31.00	1.11 (0.88-1.40)	0.387	1.41 (0.90-2.21)	0.139
**rs1982809**	**AA**		290	61.18	176	55.35	26	52.00	1	0.260	1	**0.043**
	**AG**		159	33.54	122	38.36	17	34.00	1.26 (0.94-1.71)		1.20 (0.64-2.27)	
	**GG**		25	5.27	20	6.29	7	14.00	1.32 (0.72-2.44)		3.22 (1.30-7.98)	
	**AG+GG**		184	38.82	142	44.65	24	48.00	1.27 (0.95-1.69)	0.102	1.46 (0.82-2.60)	0.207
	**AA+AG**		449	94.73	298	93.71	43	86.00	0.83 (0.45-1.50)	0.545	0.33 (0.14-0.79)	**0.014**
		**A**	739	77.95	474	74.53	69	69.00	1		1	
		**G**	209	22.05	162	25.47	31	31.00	1.21 (0.96-1.53)	0.115	1.58 (1.02-2.45)	**0.043**
**rs9288952**	**AA**		421	88.82	281	88.09	44	88.00	1	0.660	1	0.870
	**AG**		51	10.76	35	10.97	6	12.00	1.03 (0.66-1.62)		1.20 (0.50-2.86)	
	**GG**		2	0.42	3	0.94	0	0	–		–	
	**AG+GG**		53	11.18	38	11.91	6	12.00	1.08 (0.69-1.67)	0.752	1.15 (0.48-2.75)	0.862
	**AA+AG**		472	99.58	316	99.06	50	100	–		–	
		**A**	893	94.20	597	93.57	94	94.00	1		1	
		**G**	55	5.80	41	6.43	6	6.00	1.12 (0.74-1.69)	0.609	1.11 (0.48-2.56)	0.936
**rs9288953**	**CC**		188	40.00	114	36.19	16	32.00	1	0.467	1	0.542
	**CT**		218	46.38	160	50.79	26	52.00	1.21 (0.89-1.65)		1.39 (0.73-2.64)	
	**TT**		64	13.62	41	13.02	8	16.00	1.06 (0.67-1.67)		1.51 (0.63-3.61)	
	**CT+TT**		282	60.00	201	63.81	34	68.00	1.17 (0.87-1.58)	0.283	1.40 (0.75-2.58)	0.271
	**CC+CT**		406	86.38	274	86.98	42	84.00	1.05 (0.69-1.60)	0.808	0.79 (0.36-1.73)	0.643
		**C**	594	63.19	388	61.59	58	58.00	1		1	
		**T**	346	36.81	242	38.41	42	42.00	1.07 (0.87-1.32)	0.520	1.25 (0.82-1.89)	0.308
**rs1844089**	**GG**		395	83.33	265	83.86	43	86.00	1	0.366	1	0.822
	**AG**		77	16.24	47	14.87	7	14.00	0.91 (0.62-1.35)		0.88 (0.39-1.98)	
	**AA**		2	0.42	4	1	0	0	–		–	
	**AG+AA**		79	16.67	51	16.14	7	14.00	0.96 (0.66-1.42)	0.845	0.86 (0.38-1.93)	0.629
	**AG+GG**		472	99.58	312	99	50	100	–		–	
		**G**	867	91.46	577	91.30	93	93.00	1		1	
		**A**	81	8.54	55	8.70	7	7.00	1.02 (0.72-1.46)	0.913	0.85 (0.39-1.86)	0.597
**rs11921669**	**CC**		298	96.13	302	94.67	47	97.92	1	0.328	1	–
	**CT**		12	3.87	15	4.70	1	2.08	1.22 (0.57-2.62)		0.75 (0.13-4.22)	
	**TT**		0	0	2	0.63	0	0	–			
	**CT+TT**		12	3.87	17	5.33	1	2.08	1.38 (0.66-2.90)	0.384	0.75 (0.13-4.22)	0.538
	**CC+CT**		310	100	317	99.37	48	100	–		–	
		**C**	608	98.06	619	97.02	95	98.96	1		1	
		**T**	12	1.94	19	2.98	1	1.04	1.53 (0.75-3.14)	0.233	0.76 (0.14-4.22)	0.542
**rs2633582**	**AA**		265	85.76	267	83.96	43	86.00	1	0.137	1	–
	**AC**		44	14.24	47	14.78	7	14.00	1.06 (0.68-1.65)		1.03 (0.45-2.38)	
	**CC**		0	0	4	1.26	0	0	–		–	
	**AC+CC**		44	14.24	51	16.04	7	14.00	1.15 (0.74-1.78)	0.530	1.03 (0.45-2.38)	0.964
	**AA+AC**		309	100	314	98.74	50	100	–			
		**A**	574	92.88	581	91.35	93	93.00	1		1	
		**C**	44	7.12	55	8.65	7	7.00	1.23 (0.82-1.86)	0.316	1.04 (0.46-2.31)	0.966

Significant results are bolded.

**Figure 2:**
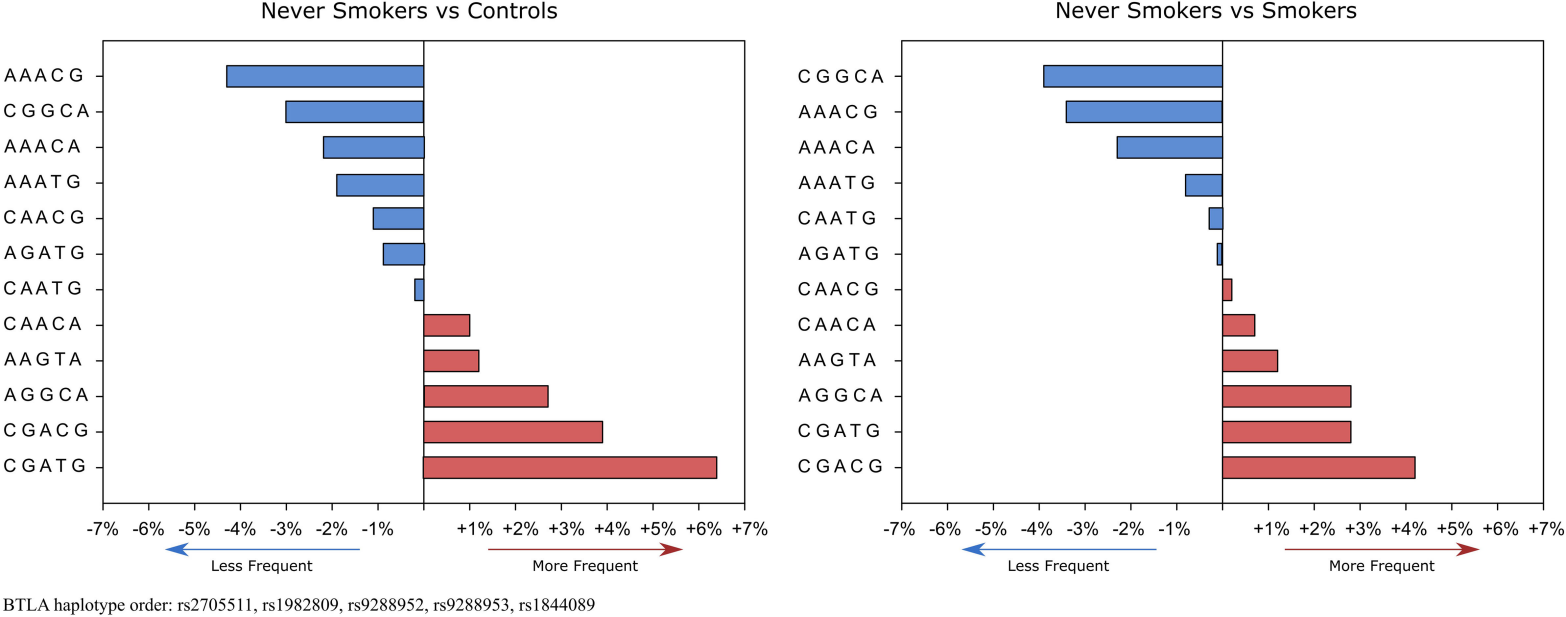
Haplotype’s frequencies distribution in never-smokers compared to controls and smokers. Red boxes show haplotypes that occur more frequently and blue boxes show haplotypes that occur less frequently in never-smokers. % values on the x-axis represent % differences in the occurrence of specific haplotypes.

Haplotype analysis revealed that global haplotype distribution differs significantly (χ2 = 37.17, df=11, p=0.0001) between never-smoking patients and controls. Additionally, haplotypes A G G C A, C G A C G, and C G A T G were more frequent in never-smoking patients than in controls (**Supplementary Table 2**). In the case of smokers, there were no differences in global haplotype distribution (**Supplementary Table 3**). However, we noticed an overrepresentation of C G A T G haplotype in smokers (15.5% vs 11.9%, OR 1.36, CI95% 1.01-1.82, p=0.042) (**Supplementary Table 4**). When comparing haplotype frequencies between smoking and never-smoking patients we observed a similar pattern of haplotype distribution as between controls and never-smoking patients (**Figure 2**).

#### 3.3.2 Analysis of *BTLA* gene variations and NSCLC risk in relation to gender

After stratification by gender, we found that the distribution of rs9288953 genotypes differs significantly between female patients and female controls (CC 27.62% vs 45.85%; CT 59.05% vs 41.46%; TT 13.33% vs 12.68%, p=0.006) and that in females the presence of rs9288953T allele (CT+TT genotypes) increased the risk of NSCLC 2.2 times (OR 2.2, CI95% 1.33-3.64, p=0.002). Moreover, we also observed that the presence of rs1982809G allele (AG+GG genotypes) increased NSCLC risk in female patients (OR 1.67, CI95% 0.99-2.55, p=0.056). In male patients, we did not notice any significant differences in alleles, genotypes, and haplotypes between patients and controls (**Table 6**).

**Table 6:** Genotypes and alleles frequencies of *BTLA* SNPs and NSCLC risk in Male and Female.

			MALE						FEMALE					
SNP	Genotype	Allele	Controls		Cases		OR (CI95%)	p value	Controls		Cases		OR (CI95%)	p value
			N	%	N	%			N	%	N	%		
**rs2705511**	**AA**		148	55.64	151	54.91	1	0.930	126	60.58	57	53.77	1	0.459
	**AC**		99	37.22	102	37.09	1.01 (0.71-1.44)		71	34.13	41	38.68	1.28 (0.78-2.09)	
	**CC**		19	7.14	22	8.00	1.13 (0.59-2.16)		11	5.29	8	7.55	1.63 (0.64-4.16)	
	**AC+CC**		118	44.36	124	45.09	1.03 (0.73-1.44)	0.865	82	39.42	49	46.23	1.32 (0.82-2.11)	0.248
	**AA+AC**		247	92.86	253	92.00	0.89 (0.47-1.67)	0.707	197	94.71	98	92.45	0.67 (0.27-1.69)	0.428
		**A**	395	74.25	404	73.45	1		323	77.64	155	73.11	1	
		**C**	137	25.75	146	26.55	1.04 (0.79-1.37)	0.767	93	22.36	57	26.89	1.28 (0.87-1.87)	0.208
**rs1982809**	**AA**		155	58.27	152	55	1	0.754	135	64.90	57	53.77	1	0.114
	**AG**		93	34.96	104	37.68	1.14 (0.80-1.63)		66	31.73	42	39.62	1.51 (0.92-2.47)	
	**GG**		18	6.77	20	7.25	1.13 (0.58-2.20)		7	3.37	7	6.60	2.36 (0.82-6.79)	
	**AG+GG**		111	41.73	124	44.93	1.14 (0.81-1.60)	0.453	73	35.10	49	46.23	1.59 (0.99-2.55)	**0.056**
	**AA+AG**		248	93.23	256	92.75	0.93 (0.49-1.79)	0.827	201	96.63	99	93.40	0.49 (0.17-1.40)	0.189
		**A**	403	75.75	408	73.91	1		336	80.77	156	73.58	1	
		**G**	129	24.25	144	26.09	1.10 (0.84-1.45)	0.486	80	19.23	56	26.42	1.51 (1.02-2.23)	**0.039**
**rs9288952**	**AA**		230	86.47	240	86.64	1	0.605	191	91.83	98	92.45	1	0.773
	**AG**		35	13.16	34	12.27	0.93 (0.56-1.54)		16	7.69	8	7.55	1.00 (0.42-2.37)	
	**GG**		1	0.38	3	1.08	–		1	0.48	0	0	–	
	**AG+GG**		36	13.53	37	13.36	0.98 (0.60-1.61)	0.952	17	8.17	8	7.55	0.94 (0.40-2.22)	0.847
	**AA+AG**		265	99.62	274	98.92	–		207	99.52	106	100	–	
		**A**	495	93.05	514	92.78	1		398	95.67	204	96.23	1	
		**G**	37	6.95	40	7.22	1.04 (0.66-1.65)	0.865	18	4.33	8	3.77	0.90 (0.39-2.05)	0.742
**rs9288953**	**CC**		94	35.47	105	38.32	1	0.774	94	45.85	29	27.62	1	**0.006**
	**CT**		133	50.19	133	48.54	0.90 (0.62-1.29)		85	41.46	62	59.05	2.34 (1.38-3.96)	
	**TT**		38	14.34	36	13.14	0.85 (0.50-1.44)		26	12.68	14	13.33	1.75 (0.82-3.76)	
	**CT+TT**		171	64.53	169	61.68	0.89 (0.62-1.26)	0.494	111	54.15	76	72.38	2.20 (1.33-3.64)	**0.002**
	**CC+CT**		227	85.66	238	86.86	1.11 (0.68-1.80)	0.686	179	87.32	91	86.67	0.93 (0.47-1.85)	0.872
		**C**	321	60.57	343	62.59	1		273	66.59	120	57.14	1	
		**T**	209	39.43	205	37.41	0.89 (0.72-1.17)	0.495	137	33.41	90	42.86	1.49 (1.06-2.10)	**0.021**
**rs1844089**	**GG**		215	80.83	225	82.12	1	0.337	180	86.54	95	89.62	1	0.613
	**AG**		50	18.80	45	16.42	0.86 (0.55-1.34)		27	12.98	11	10.38	0.79 (0.38-1.64)	
	**AA**		1	0.38	4	1.46	–		1	0.48	0	0	–	
	**AG+AA**		51	19.17	49	17.88	0.92 (0.60-1.42)	0.700	28	13.46	11	10.38	0.76 (0.37-1.58)	0.434
	**AG+GG**		265	99.62	270	98.54	–		207	99.52	106	100	–	
		**G**	480	90.23	495	90.33	1		387	93.03	201	94.81	1	
		**A**	52	9.77	53	9.67	0.99 (0.66-1.48)	0.955	29	6.97	11	5.19	0.75 (0.37-1.51)	0.387
**rs11921669** ** **	**CC**		174	96.67	260	94.20	1	0.346	123	95.35	102	97.14	1	–
	**CT**		6	3.33	14	5.07	1.49 (0.58-3.84)		6	4.65	3	2.86	0.65 (0.17-2.44)	
	**TT**		0	0	2	0.72	–		0	0	0	0	–	
	**CT+TT**		6	3.33	16	5.80	1.70 (0.67-4.30)	0.231	6	4.65	3	2.86	0.65 (0.17-2.44)	0.479
	**CC+CT**		180	100	274	99.28	–		129	100	105	100	–	
		**C**	354	98.33	534	96.74	1		252	97.67	207	98.57	1	
	** **	**T**	6	1.67	18	3.26	1.89 (0.76-4.66)	0.142	6	2.33	3	1.43	0.66 (0.18-2.43)	0.483
**rs2633582**	**AA**		153	85.00	227	82.25	1	0.244	111	86.05	95	89.62	1	–
	**AC**		27	15.00	45	16.30	1.12 (0.67-1.87)		18	13.95	11	10.38	0.73 (0.33-1.59)	
	**CC**		0	0	4	1.45	–		0	0	0	0	–	
	**AC+CC**		27	15.00	49	17.75	1.21 (0.73-2.02)	0.441	18	13.95	11	10.38	0.73 (0.33-1.59)	0.408
	**AA+AC**		180	100	272	98.55	–		129	100	106	100	–	
		**A**	333	92.50	499	90.40	1		240	93.02	201	94.81	1	
		**C**	27	7.50	53	9.60	1.3 (0.80-2.10)	0.273	18	6.98	11	5.19	0.74 (0.35-1.59)	0.423

Significant results are bolded.

#### 3.3.3 Analysis of *BTLA* gene variations and NSCLC risk in relation to histological type

After stratification by NSCLC histological type, we did not observe any significant changes in genotypes distributions. However, we noticed a shift in genotype frequencies in patients with adenocarcinoma (AD) for SNPs rs1982809 (AA 53% vs 61%; AG 39% vs 33.5%; GG 8% vs 5%) and rs2705511 (AA 49.5% vs 58%; AC 41% vs 36%; CC 9% vs 6%) comparing to controls. Rs1982809G allele carriers (AG+GG genotypes) and rs2705511C allele carriers (AC+CC genotypes) were more frequent in AD patients compared to healthy controls (46.85% vs. 38.82% and 50.45% vs. 42.19%, respectively). This shift was not significant probably due to the too-small size of NSCLC subgroups (**Supplementary Table 5**).

#### 3.3.4 Analysis of *BTLA* gene variations and NSCLC risk in relation to stage of disease

**Figure 3:**
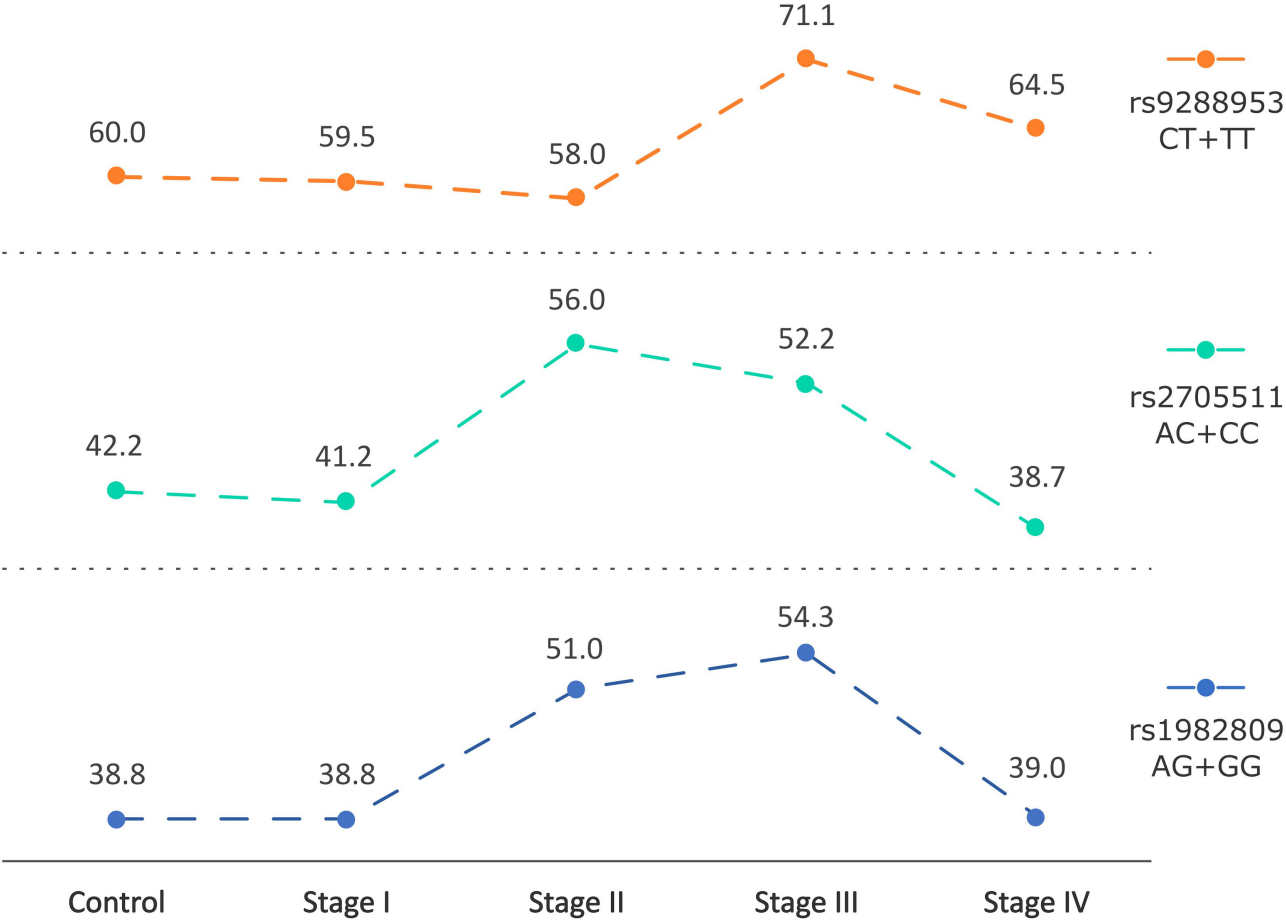
Changes in frequencies of rs2705511C, rs1982809G, and rs9288953T alleles carriers based on the stage of NSCLC. Points indicate % frequency of selected SNPs in the specific stage of NSCLC.

Taking into consideration the stage of disease in NSCLC patients we observed that rs1982809 and rs2705511 correlated with the more advanced stages of NSCLC (stage II and III), but not with the most advanced stage IV. Allele distribution analysis showed overrepresentation of rs1982809G and rs2705511C alleles in patients at stage II compared to controls (31.4% vs 22%, p=0.03 and 33% vs 24.3%, p=0.055, respectively). Furthermore, genotype frequency analysis showed that the distribution of the rs1982809, rs270511, and rs9288953 genotypes differed significantly between stage III patients and controls (p=0.007, p=0.04 and p=0.02, respectively) (**Figure 3**). What’s more, the presence of rs1982809G allele (AG+GG genotypes), and rs2705511C allele (AC+CC genotypes) and rs9288953T (CT and TT genotypes) were more frequent in these patients (54.3% vs 38.8%, p=0.0025; 52.2% vs 42.2%, p=0.053; 71% vs 60%, p=0.03, respectively) in comparison to controls (**Supplementary Table 6**).

### 3.4 *BTLA* gene polymorphisms in relation to overall survival

The polymorphic features of *BTLA* as well as gender, age, stage of disease, histological type, smoking status, surgery, or lack of it, were subjected to overall survival (OS) analysis. The analysis performed in the whole group of NSCLC patients indicated that as previously (34): gender, stage of disease, surgery, and cigarette smoking significantly influenced OS. As was shown in **Supplementary Figure 1**, OS in males was significantly shorter than in females (mean ± SD: 28.7 ± 37.3 vs. 40.0 ± 40.4, p=0.002). Similarly, to earlier results, surgery treatment significantly increased OS in comparison to no surgery (mean ± SD: 52.1 ± 42.4 vs. 8.52 ± 9.93, p<0.001). As expected, the clinical stage of disease at the time of diagnosis was strongly related to OS, in detail, mean OS was 61.3, 42.2, 27.1 and 7.0 for stages I, II, III and IV, respectively (p<0.001). Since cigarette smoking is a predominant NSCLC risk factor, we noticed that smoking *per se* decreased OS, while heavy smoking (>31 pack-years) dramatically shortened OS (**Supplementary Figure 1**). What’s more, higher pack-years increased the risk of death by 2% (HR 1.02 95%CI 1.01-1.03, p<0.001) (**Supplementary Figure 2**).

**Figure 4:**
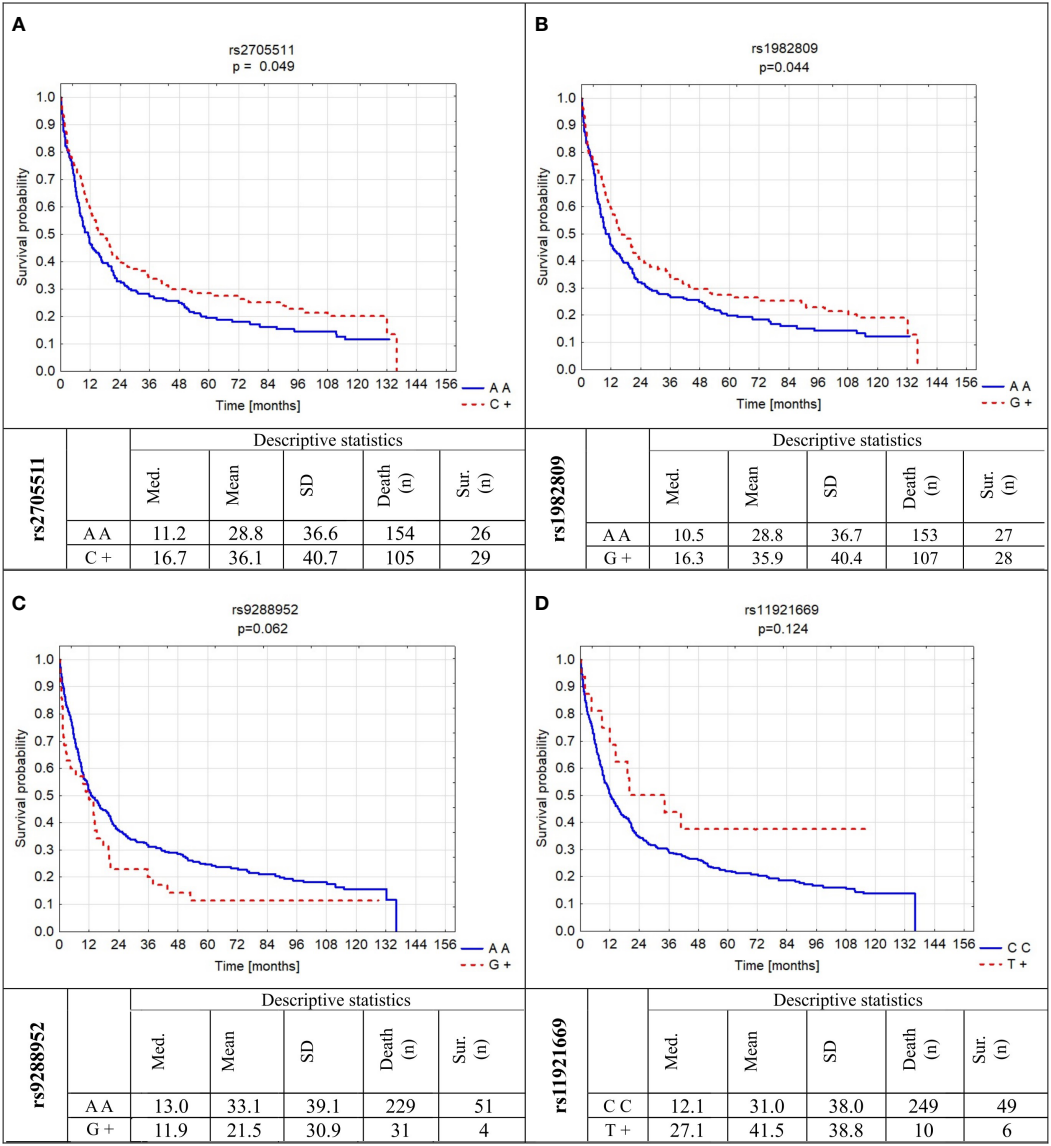
Probability of survival in relation to *BTLA* gene polymorphisms: **(A)** rs2705511; **(B)** rs1982809; **(C)** rs9288952; **(D)** rs11921669. Med., median; SD, standard deviation; sur., survival; n, number of patients.

Besides known OS influencing factors, we also analyzed the relation of *BTLA* SNPs investigated here to OS. We found out that two of them significantly modified OS and two others have a tendency to be associated with OS (**Figure 4**). In detail, the presence of C allele for rs2705511 extended OS as compared to AA homozygotes for more than half a year (mean ± SD: 36.1 ± 40.7 vs. 28.8 ± 36.6, p=0.049). Similarly, patients carrying G allele in rs1982809 lived about six months longer than their AA counterparts (mean ± SD: 35.9 ± 40.4 vs. 28.8 ± 36.7, p=0.044). For rs9288952, we noticed a borderline tendency to shorter OS for patients possessing G allele as compared to AA individuals (**Figure 4**). Moreover, we noticed no statistically significant association of homozygosity CC in rs11921669 with longer OS as compared to patients possessing T allele (**Figure 4**). The results of univariate Cox analysis of OS for all factors are presented in **Supplementary Table 7**.

Next, we analyzed the influence of mentioned above factors on OS in subgroups of never-smoking and smoking patients. In never-smoking patients only stage of disease and surgery treatment were significantly associated with OS (**Supplementary Table 7**). Interestingly, in smoking patients, despite of male gender, IV stage of disease, and lack of surgery also rs2705511AA genotype and rs1982809AA genotype were significantly associated with the death risk (HR 1.45 and 1.38, respectively). Similarly, as described for all NSCLC patient’s duration of smoking increased the risk of death (**Supplementary Table 7**).

### 3.5 Multivariate analysis of risk factors influence on survival of NSCLC patients

In the multivariate analysis in the model which included rs2705511, rs1982809, rs9288952, rs11921669, gender, stage of disease, surgery or no surgery, smoking, and pack-years indicator, the following parameters: rs1982809 AA genotype, possessing of rs9288952G allele, lack of surgery, IV stage of disease, and pack years were independent increased risk of death factors in NSCLC patients (**Table 7**). When the analysis was performed separately for smokers, in similar to the univariate analysis stage of NSCLC, surgery, rs1982809, and rs9288952 were independent risk factors for OS (**Table 7**).

**Table 7:** Multivariate Cox analysis of factors influencing OS for whole group of NSCLC patients and Smoking NSCLC group of patients.

		Whole group of NSCLC patients				Smoking NSCLC patients			
Factor		Coefficient	p value	HR	95% CI	Coefficient	p value	HR	95% CI
rs1982809	A A	**0.17**	**0.02**	**1.41**	**1.06-1.89**	**0.21**	**0.01**	**1.52**	**1.12-2.07**
rs9288952	A A	**-0.32**	**0.00**	**0.53**	**0.34-0.81**	**-0.31**	**0.01**	**0.53**	**0.33-0.86**
rs11921669	C C	–	–	–	–	–	–	–	–
Gender	M	–	–	–	–	–	–	–	–
Pack-years		**0.01**	**0.026**	**1.01**	**1.00-1.02**	–	–	–	–
Clinical stage	II	-0.29	0.07	0.63	0.36-1.11	-0.21	0.20	0.69	0.39-1.22
	III	0.12	0.30	2.00	1.31-3.05	0.04	0.72	1.75	1.12-2.73
	IV	**0.74**	**0.00**	**3.74**	**2.25-6.21**	**0.68**	**0.00**	**3.29**	**1.92-5.63**
Surgery	yes	**-0.50**	**0.00**	**0.37**	**0.24-0.55**	**-0.57**	**0.00**	**0.32**	**0.21-0.49**

HR – hazard ratio, 95% CI – confidence interval. Significant results are bolded.

## 4 Discussion

BTLA is an essential immune checkpoint molecule in regard to autoimmune diseases (18, 19), infections (20, 21), and cancer (22, 23). The expression of BTLA and its ligand HVEM has been documented to be deregulated in cancer (15, 22, 25, 39). It was shown, in murine models, that BTLA blockade, alone or in combination with chemotherapy, led to improvement of survival in epithelial ovarian carcinoma (12). In melanoma, where BTLA is highly expressed on tumor-specific CD8+ T cells, its blockade resulted in enhanced proliferation and cytokine production (40). In gastric cancer, high expression of BTLA correlates with a poor prognosis on OS as well as with lymph node metastasis (41). What’s more, BTLA expression was found on tumor cells in lung adenocarcinoma patients (42) as well as on tumor cells in gastric cancer (43). In several solid tumors and also in hematological malignances levels of soluble BTLA in serum correlated with disease prognosis (44–47). Therefore, it is not surprising, that associations of its SNPs with several neoplasms were described (24, 31), including NSCLC in Tunisian (33) and Chinese populations (32).

Our previous studies on *BTLA* SNPs in cancer showed an association of rs1982809, rs2705511, and rs9288953 with chronic lymphocytic leukemia (CLL) risk (24). Moreover, we found that rs1982809 and rs2705511 might be considered low-penetrating risk factors for renal cell cancer development (31). Both studies were conducted on the Caucasian population. Also, studies of other groups of the Chinese population showed a correlation between *BTLA* SNPs and cancer development. In one of the first works on *BTLA* SNPs in cancer, Fu’s group reported that rs1844089[CT] and rs2705535[CT] genotypes increased the risk of breast cancer while rs1844089 [CC], rs2705535[CC] and rs9288952 [GG] genotypes decreased its risk. Haplotype analysis showed correlation of haplotype G T T T T (rs9288952, rs2931761, rs2633562, rs2705535, rs1844089) with susceptibility to breast cancer (26). Ge et al. found that the rs2705535[TT] genotype is associated with the risk of rectal cancer while the presence of rs9288953 [TT] genotype lowered its risk (27). On contrary Cao et al. did not observe any significant relationship between *BTLA* polymorphisms and esophageal squamous cell carcinoma (29). Similarly, also Tang et al. did not report differences in genotype frequencies between esophagogastric junction adenocarcinoma (EGJA) cases and controls in overall comparison, however, haplotype analysis revealed that haplotype T A A G (rs16859629, rs1982809, rs2171513, rs31122708) increased risk of EGJA development. Additionally, in subgroup analysis, they observed that in smokers rs1982809G>A was associated with increased susceptibility to EGJA (28). All of these studies were conducted on the Chinese population and there is no data for Caucasians.

Here, we found an association of rs1982809 SNP (G allele and AG + GG genotypes) with a risk of NSCLC in Poles. Similarly, Khadhraoui et al. documented an association of rs1982809 with an increased risk of lung cancer. They found similarly to us that frequencies of the genotypes AG and GG were significantly overrepresented among lung cancer patients compared to controls (33). On the contrary in the Chinese population, Wang et al. observed that compared to rs1982809 GG genotype carriers of A allele (AA+AG genotypes) had an increased risk of the development of NSCLC (32). That difference may result from big differences in rs1982809 frequency between these two populations where in the majority of populations including Caucasians the distribution of alleles is similar (A=60-77%; G=40-23%) and only in the Asian population (but not in the South Asian) the frequency of alleles is in opposite with overexpression of G allele (A=25-35%; G=75-65%) (based on dbSNP database). Other tested here *BTLA* SNPs did not reveal associations with NSLC risk. This may be explained by a lack of strong LD between rs1982809 and other SNPs in our population. Among *BTLA* haplotypes consisting of five tested SNPs (rs270551, rs1982809, rs9288952, rs9288953, rs1844089), only one (C G A T G) was associated with NSCLC, likely because of possessing rs1982809G allele. Other haplotypes containing this allele were too infrequent to reach statistical association. Interestingly, when we divided our patients into smokers and never-smokers, the association of the rs1982809G allele with NSCLC risk was detectable only in never-smokers but not in smokers. It should be emphasized here that the group of never-smokers was much smaller than smokers, but still, the association was visible only in the former group. Also, *BTLA* haplotypes were distributed differently in never-smokers than in smokers and controls, whereas there was no difference between these two latter groups. These results might be interpreted as caused by a high representation of smokers in our control group (for a majority of which we had no information on their smoking status, unfortunately). The prevalence of smoking in Poland in 2019 was 26% of current smokers (plus 25.8% of former smokers (48); smoking cessation decreases lung cancer risk to some extent, but not completely (2)). This might have been a cause that the frequencies of rs1982809 alleles and genotypes in our control group and in smoking patients were similar. Also, the explanation of a lack of *BTLA* SNPs effect on smokers might lay in such a strong contribution of smoking to cancer risk that a relatively weak effect of rs1982809 disappeared. In never-smokers this strong carcinogen is lacking, paving the way for genetic factors. It is already known that although lung cancer in smokers has frequently more mutations than in never-smokers, most of them are “passenger” mutations, whereas causative mutations prevail in never-smokers (49), and also distinct germ-line polymorphisms reveal an association with lung cancer in smokers and never-smokers (50). Indeed, we recently described distinct associations of SNPs in endoplasmic reticulum aminopeptidase 1 (ERAP1) gene with NSCLC in smokers versus never-smokers, in both groups different from controls for the majority of SNPs, and mostly in opposite directions (34). In that case, the detection of the effects of *ERAP1* SNPs also in smokers may be explained by the contribution of peptide trimming by this enzyme to production of tumor antigenic epitopes different in smokers and never-smokers. Furthermore, the correlation of patients smoking status with the frequency of *BTLA* SNPs was also observed in esophagogastric junction adenocarcinoma (EGJA). Tang et al. noticed that rs1982809G>A was associated with increased susceptibility to EGJA in ever-smokers (28).

The finding that rs9288953T allele was associated with cancer only in women but not in men may result from an almost three-fold higher fraction of never-smokers and a four-fold lower fraction of heavy smokers (>40 pack-years) in women. Thus, a strong carcinogen (smoking) masked the genetic effect of BTLA in women to a lesser extent than in men. It should be emphasized here that women were twice less numerous among our patients, but still, the SNP association was found only there, where they were not diluted by a twice higher number of men who did not display such association. On the other hand, polymorphisms in several genes were observed to affect lung cancer risk in women, while others contributed to the risk in males (51). In addition, the association of sex hormones including estrogen with carcinogenesis has been well established. Multiple studies have shown a correlation between estrogen and its receptor’s expression levels with lung cancer pathogenesis and progression (52). Polymorphism rs9288953C>T is situated in the first intron of *BTLA* gene, where according to literature (53) SNPs located within the first intron can modify the splicing process and may regulate the gene expression. Ge et al. (27) postulated, based on the human splicing finder software, that rs9288953C>T SNP could activate six new splice sites in splicing enhancer motifs and break one splicing sites in the silencer motif and in this way may enhance the splicing signal and strengthen the expression of BTLA. In the colorectal cancer patients Ge et al. observed that carriers of rs9288953T allele (CT+TT genotypes) had increased susceptibility of colorectal cancer (27). Therefore, our result with *BTLA* polymorphisms, if confirmed on a much larger cohort of smoking and never-smoking patients and controls of both sexes separately, may add a new element to molecular and immunological differences between women and men suffering from lung cancer, depending on their smoking status.

Interestingly, rs2705511 together with rs1982809 were associated with stage II and stage III of cancer (and rs9288953 with stage III). The reason why these SNPs lost association in stage IV is obscure. This is difficult to explain why *BTLA* SNPs are associated with II and III stages of the disease but not IV stage since this parameter depends on many factors, among others availability of medical services and personal motivation for regular health examinations. However, it might be somehow connected with the progression rate and metastasis capacity of the tumor. The immune defense risk factor association we observed disappeared in the late stages of the disease, which can be explained by the interruption of anti-tumor defenses and the full development of the tumor influenced by other factors related to the nature of cancer.

Another interesting funding is a tendency for overrepresentation of rs1982809 and rs2705511 alleles in association with NSCLC histological type. rs1982809G and rs2705511C carriers were more frequent among adenocarcinoma (AD) patients than in controls. Similarly, Khadhraoui et al. observed that the presence of at least one copy of rs1982809G allele (AG+GG genotypes) was correlated with AD risk but not with SCC risks (33). The predominance of AD patients among never-smokers has been well documented earlier by others (54, 55). In one of those studies, Santoro’s group showed that among all never-smoking lung cancer patients 70% of them developed adenocarcinoma and most of them were women (54). To this date, several genetic lesions have been reported to have an influence on adenocarcinoma development in never-smoking patients. For example, Mandour et al. (56) documented that SNPs within telomerase reverse transcriptase (TERT), cleft lip, and palate transmembrane 1-like (CLPT-M1L) genes can have an important role in the development of adenocarcinoma in never-smokers. In another study Staff et al. (57) showed that gene expression profile differs between AD patients who never smoked and those who smoked suggesting a distinct entity of lung adenocarcinomas in those two groups. Therefore, it is not surprising that rs1982809 and rs2705511 SNPs could potentially be related to adenocarcinoma development in never-smoking patients, however further studies are needed on a larger group to confirm this suspicion.

In the present work, we also analyzed in univariate and multivariate analysis the factors influencing OS. As was expected and shown in a previous paper (34) several clinical factors like: female gender, less than III clinical stages of the disease and surgery vs other treatment were associated with better overall survival. As was shown by us and many others (2, 54, 55), smoking has influence on OS in NSCLC patients. Especially, heavy smoking (>40 pack-years) increased the risk of death about 4 times (HR 3.76). Moreover, here we showed that also intensity of smoking (pack-years) influenced OS. Although the increased HR is only 2% per year however long exposition to cigarette smoking significantly influences OS (p<0.001).

What’s more, we noticed an association of variations within *BTLA* gene with OS. What’s interesting factors related to the risk of NSCLC: rs1982809 SNP (G allele and AG+GG genotypes) together with being in moderate LD rs270551 (possessing of C allele (AC+CC genotypes)) were associated with better survival of NSCLC patients. It is difficult to explain why the factors associated with better survival increased the risk of NSCLC, since relatively little is known about BTLA expression in particular subpopulations of immune cells and about the function of these SNPs. Therefore, it is hard to speculate about the role of BTLA in carcinogenesis, metastasis, and disease progression. Nevertheless, it is known that BTLA is highly expressed on anergic cells, and the lower potency of rs1982809 G allele (24) to produce BTLA mRNA might be associated with anergy stage (58). On the other hand, it is known that BTLA is a marker of T cell exhaustion. However, it might be associated with late stages of T cell exhaustion since as it is postulated that IC expression is sequential and begins from PD-1, then TIM-3, CTLA-4, LAG3, and finally BTLA expression (59). Furthermore, there is only one study describing BTLA tissue expression in NSCLC samples, where authors showed a significant negative correlation between BTLA expression and OS (42). In this context, our recent work showing higher expression of BTLA mRNA in rs1982809AA individuals (24) may explain the higher risk of death in these individuals.

On the other hand, both SNPs (rs2705511 and rs1982809) are situated between genes encoding CD200 and BTLA while rs1982809 is placed in a 3′ nearby gene region of *BTLA* (−101,081||−73 bp) and rs2705511 is located in their intragenic region (−97,820 bp||−3334 bp). CD200 has been shown to play an important role in the regulation of anti-tumor immunity, and overexpression of CD200 has been reported in a number of hematological malignancies and solid tumors as well as on cancer stem cells (60–62). Therefore, the role of CD200 in carcinogenesis and progression of cancer is not unlikely. Additionally, according to our preliminary analysis, miR-511-3p may bind in the place of occurrence of rs1982809, therefore the presence of rs1982809 G allele could possibly influence on miR-511-3p binding. Nevertheless, it is not known if the expression of miR-511-3p is present and/or dysregulated in NSCLC, however, it is been shown that miR-511-3p is significantly downregulated in prostate cancer cell lines, and human prostate tumors (63). In the case of rs270551 potential role in the regulation of BTLA and/or CD200 expression based on UCSC database we noticed that rs2705511 is located in a possible transcription factor binding region for Runx1. However, there is no study confirming this interaction. Published knowledge about genetic and epigenetic mechanisms regulating BTLA expression is very limited, especially about the functional role of BTLA polymorphisms. Therefore, it is hard to speculate on that subject.

We also noticed a strong trend for the association of rs9288952 with OS, but not with risk of disease. In the study by Fu et al. (26) who investigated the association between the *BTLA* SNPs: rs1844089, rs2705535, rs9288952, rs2633562, and rs2931761 it was noticed that among others rs9288952 was related to the risk of malignant breast cancer in Chinese women and its clinical features like tumor size, estrogen and progesterone receptor expression as well as C-erbB and P53 status. rs9288952 is located within exon 5 of *BTLA* gene which causes substitution of A to G nucleotide resulting in missense mutation which consequently causes a change in amino acid from proline (P) into leucine (L) (P267L). Both amino acids belong to non-polar aa’s, however, it’s been shown that this kind of mutation may have an influence on protein structure and protein-protein/protein-DNA interactions. Dansault et al. showed that L46P mutation within PAX6 gene is located in the DNA-binding paired-domain what may affect helix-turn-helix motif, and have an influence on the DNA-binding properties (64). Exon 5 of *BTLA* gene together with exon 4 create a cytoplasmic tail of BTLA protein. Therefore, the scenario in which P267L exchange caused by the presence of rs9288952 has an influence on protein properties and downstream signaling cannot be excluded without further studies. Here we found that rs9288952 possession of G allele increased the risk of death by 50% suggesting an important role of this SNP in NSCLC progression.

In the end, we observed a tendency for better survival in patients carrying T allele in rs11921669 SNP. We selected this SNP since our *in silico* analysis showed that rs11921669, a 2kb upstream variant, can be located in a potential regulatory region of *BTLA* gene. According to the RegulomeDB (65) the Rank for this SNP is 0.85 (Rank=1 is the maximum score, which designates variants with the highest probability to be regulatory SNPs) suggesting that it may have functional relevance. Further, in depth-studies are needed to explain the relationship observed by us between the presence of allele T and better survival of NSCLC patients.

The results of multivariate Cox analysis of the factor influencing overall survival which included the following BTLA SNPs: rs2705511, rs1982809, rs9288952, rs11921669 as well as gender, stage of disease, surgery or no surgery, smoking, and pack-years indicator confirmed the results of univariate analysis. In details, the results indicated that in the whole group of NSCLC patients as well as in smoking NSCLC patients rs1982809 AA genotype, possessing of rs9288952G allele, lack of surgery, higher stages of the disease, and higher pack years indicator are independent factors increasing the risk of death.

The limitation of our study is a low number of nonsmoking patients, despite the quite big overall number of patients. As was mentioned before the majority of NSCLC patients are addicted to smoking, which is why the number of nonsmoking patients is low. The next limitation is the lack of information about smoking habits among controls. The control group was recruited in different time periods, from blood bank volunteer donors and data about smoking were not available for the study. However, the strength of this study is a long period of observations lasting more than 10 years.

## 5 Conclusions

In conclusion, our study showed an association of *BTLA* polymorphisms with NSCLC risk. Especially rs1982809 may be considered a low penetrating risk factor for the development of NSCLC. Moreover, we found genetic associations of *BTLA* rs1982809 polymorphism with NSCLC in never-smokers (where genetics may play a much stronger role in addition to environmental factors other than tobacco) but not in smokers (where tobacco smoke is the strongest factor). However, an extended study on a larger group of never-smoking patients is needed to confirm our results. Additionally, there was evidence suggesting that variants of rs27055011 and/or rs9288953 may also relate to the risk of NSCLC in females, and adenocarcinoma development. Furthermore, rs1982809 and rs9288952 were found to be together with male gender, heavy smoking, advanced stage of disease, and lack of surgery, an independent risk factor for patients’ survival suggesting the influence of *BTLA* polymorphisms on disease progression. Nevertheless, studies with larger patients’ group and functional evaluation of studied *BTLA* polymorphisms are needed to confirm our findings.

## Data availability statement

The datasets presented in this study can be found in the article/**Supplementary Material**, further inquiries can be directed to the corresponding author.

## Ethics statement

The studies involving human participants were reviewed and approved by Bioethical Committee of Wrocław Medical University, Wrocław, Poland. The patients/participants provided their written informed consent to participate in this study.

## Author contributions

LK and MJ conceived and designed the experiments. AA and AP performed the experiments. AA and KPt performed statistical analysis. IP and KP contributed to patients’ recruitment and clinical characteristics. AW contributed to controls recruitment. AA and LK wrote the paper. PK, LK and AA wrote the Discussion. MJ provided finance support. All authors contributed to the article and approved the submitted version.
