# Supplementary material for: The association of BTLA gene polymorphisms with non-small lung cancer risk in smokers and never-smokers
Source: Front Immunol. 2023 Jan 19;13:1006639. doi: 10.3389/fimmu.2022.1006639 (PMC9893504; doi:10.3389/fimmu.2022.1006639)
Supplement: Supplementary file 1 [file DataSheet_1.docx]

**Supplementary materials**

**Table 1** Characteristics of BTLA SNPs

| **Chr.** | **Gene** | **SNP** | **Variation** | **Gene region** | **Consequence** |
| --- | --- | --- | --- | --- | --- |
| 3 | *BTLA* | rs1982809 | A>G | 3’UTR | Downstream Transcript Variant |
| 3 | *BTLA* | rs2705511 | A>C | 3’UTR | Downstream Transcript Variant |
| 3 | *BTLA* | rs9288952 | A>G, P267L | Exon 5 | Missense Variant |
| 3 | *BTLA* | rs9288953 | C>T | Intron 1 | Intron Variant |
| 3 | *BTLA* | rs1844089 | G>A | Intron 1 | Intron Variant |
| 3 | *BTLA* | rs2633582 | A>C | 5’UTR | Upstream Transcript Variant |
| 3 | *BTLA* | rs11921669 | C>T | 5’UTR | Upstream Transcript Variant |

**Table 2** Haplotype frequencies in never smokers and controls

| **Haplotype*** | **Never Smokers** | **Control** | **Odds Ratio** [95%CI] | **p value** |
| --- | --- | --- | --- | --- |
| **A A A C A** | 0.0 | 2.2 | - | 0.136 |
| **A A A C G** | 43.6 | 47.9 | 0.813 [0.534~1.239] | 0.335 |
| **A A A T G** | 19.4 | 21.3 | 0.868 [0.514~1.468] | 0.598 |
| **A A G T A** | 1.2 | 0.0 | - | 0.001 |
| **A G A T G** | 1.0 | 1.9 | 0.549 [0.074~4.060] | 0.551 |
| **A G G C A** | 4.2 | 1.5 | 2.798 [0.915~8.552] | 0.060 |
| **C A A C G** | 3.0 | 4.1 | 0.710 [0.213~2.363] | 0.574 |
| **C A A T G** | 1.2 | 1.4 | 0.870 [0.132~5.737] | 0.885 |
| **C G A C G** | 6.4 | 2.5 | 2.607 [1.050~6.473] | 0.032 |
| **C G A T G** | 18.3 | 11.9 | 1.624 [0.938~2.813] | 0.081 |
| **C G G C A** | 0.0 | 3.0 | - | 0.081 |
| **C A A C A** | 1.0 | 0.0 | - | 0.002 |
| **Total** | 99.3% | 97.7% |  |  |
|  | Global χ2=37.17, df=11, **p=0.000112** | |  |  |
| * BTLA haplotype order: rs2705511, rs1982809, rs9288952, rs9288953, rs1844089 | | | | |

**Table 3** Global haplotype distribution between smoking patients and controls

| **Haplotype*** | **Smokers** | **Control** | **Odds Ratio** [95%CI] | **p value** |
| --- | --- | --- | --- | --- |
| **A A A C A** | 2.3 | 2.2 | 1.055 [0.533~2.089] | 0.877 |
| **A A A C G** | 47.0 | 47.9 | 0.960 [0.782~1.179] | 0.697 |
| **A A A T G** | 20.2 | 21.3 | 0.932 [0.725~1.199] | 0.585 |
| **A G A T G** | 1.1 | 1.9 | 0.592 [0.244~1.438] | 0.242 |
| **A G G C A** | 1.4 | 1.5 | 0.938 [0.403~2.184] | 0.882 |
| **C A A C G** | 2.8 | 4.1 | 0.658 [0.369~1.173] | 0.153 |
| **C A A T G** | 1.5 | 1.4 | 1.077 [0.460~2.525] | 0.864 |
| **C G A C G** | 2.2 | 2.5 | 0.868 [0.441~1.709] | 0.682 |
| **C G A T G** | 15.5 | 11.9 | 1.356 [1.011~1.820] | 0.042 |
| **C G G C A** | 3.9 | 3.0 | 1.307 [0.749~2.278] | 0.345 |
| **Total** | 97.9% | 97.7% |  |  |
|  | Global χ2=8.31, df=9, p=0.503 | |  |  |

* BTLA haplotype order: rs2705511, rs1982809, rs9288952, rs9288953, rs1844089

**Table 4** Global haplotype distribution between non-smoking and smoking patients

| **Haplotype*** | **Never Smokers** | **Smokers** | **Odds Ratio** [95%CI] | **p value** |
| --- | --- | --- | --- | --- |
| **A A A C A** | 0.0 | 2.3 | - | 0.126 |
| **A A A C G** | 43.6 | 47.0 | 0.851 [0.553~1.310] | 0.464 |
| **A A A T G** | 19.4 | 20.2 | 0.934 [0.545~1.601] | 0.805 |
| **A A G T A** | 1.2 | 0.0 | - | 0.007 |
| **A G A T G** | 1.0 | 1.1 | 0.930 [0.116~7.465] | 0.945 |
| **A G G C A** | 4.2 | 1.4 | 2.990 [0.910~9.824] | 0.059 |
| **C A A C A** | 1.0 | 0.3 | 3.947 [0.480~32.450] | 0.126 |
| **C A A C G** | 3.0 | 2.8 | 1.082 [0.308~3.795] | 0.902 |
| **C A A T G** | 1.2 | 1.5 | 0.810 [0.119~5.513] | 0.829 |
| **C G A C G** | 6.4 | 2.2 | 3.010 [1.136~7.979] | 0.020 |
| **C G A T G** | 18.3 | 15.5 | 1.201 [0.688~2.096] | 0.519 |
| **C G G C A** | 0.0 | 3.9 | - | 0.047 |
| **Total** | 99.3% | 98.2% |  |  |
|  | Global χ2=24.2, df=11, p=0.012 | |  |  |
| * BTLA haplotype order: rs2705511, rs1982809, rs9288952, rs9288953, rs1844089 | | | | |

**Table 5** Distribution of genotypes and alleles frequencies of BTLA SNPs according to histological type

|  |  |  | **Controls** | | **Squamous Cell Carcinoma (SqCC)** | | | | **Adenocarcinoma (AD)** | | | |
| --- | --- | --- | --- | --- | --- | --- | --- | --- | --- | --- | --- | --- |
| **SNP** | **Genotype** | **Allele** | **N** | **%** | **N** | **%** | **OR (CI95%)** | **p value** | **N** | **%** | **OR (CI95%)** | **p value** |
| rs2705511 | **AA** |  | 274 | 57.81 | 67 | 57.26 | 1 | 0.683 | 55 | 49.55 | 1 | 0.248 |
|  | **AC** |  | 170 | 35.86 | 40 | 34.19 | 0.97 (0.63-1.50) |  | 46 | 41.44 | 1.35 (0.87-2.08) |  |
|  | **CC** |  | 30 | 6.33 | 10 | 8.55 | 1.40 (0.66-2.96) |  | 10 | 9.01 | 1.70 (0.80-3.63) |  |
|  | **AC+CC** |  | 200 | 42.19 | 50 | 42.74 | 1.02 (0.68-1.54) | 0.916 | 56 | 50.45 | 1.39 (0.92-2.11) | 0.115 |
|  | **AA+AC** |  | 444 | 93.67 | 107 | 91.45 | 0.70 (0.34-1.46) | 0.393 | 101 | 91 | 0.66 (0.32-1.38) | 0.314 |
|  |  | **A** | 718 | 75.74 | 174 | 74.36 | 1 |  | 156 | 70.27 | 1 |  |
|  |  | **C** | 230 | 24.26 | 60 | 25.64 | 1.08 (0.78-1.50) | 0.661 | 66 | 29.73 | 1.32 (0.96-1.83) | 0.092 |
| rs1982809 | **AA** |  | 290 | 61.18 | 67 | 57.26 | 1 | 0.239 | 59 | 53.15 | 1 | 0.233 |
|  | **AG** |  | 159 | 33.54 | 39 | 33.33 | 1.07 (0.69-1.65) |  | 43 | 38.74 | 1.33 (0.86-2.06) |  |
|  | **GG** |  | 25 | 5.27 | 11 | 9.40 | 1.94 (0.92-4.09) |  | 9 | 8.11 | 1.82 (0.82-4.03) |  |
|  | **AG+GG** |  | 184 | 38.82 | 50 | 42.74 | 1.18 (0.78-1.77) | 0.438 | 52 | 46.85 | 1.39 (0.92-2.10) | 0.120 |
|  | **AA+AG** |  | 449 | 94.73 | 106 | 90.60 | 0.53 (0.25-1.09) | 0.095 | 102 | 91.89 | 0.61 (0.28-1.33) | 0.250 |
|  |  | **A** | 739 | 77.95 | 173 | 73.93 | 1 |  | 161 | 72.52 | 1 |  |
|  |  | **G** | 209 | 22.05 | 61 | 26.07 | 1.25 (0.90-1.74) | 0.190 | 61 | 27.48 | 1.34 (0.97-1.87) | 0.08 |
| rs9288952 | **AA** |  | 421 | 88.82 | 106 | 89.83 | 1 | 0.764 | 99 | 89.2 | 1 | 0.791 |
|  | **AG** |  | 51 | 10.76 | 12 | 10.17 | 0.96 (0.50-1.85) |  | 12 | 10.81 | 1.03 (0.53-1.98) |  |
|  | **GG** |  | 2 | 0.42 | 0 | 0 | - |  | 0 | 0 | - |  |
|  | **AG+GG** |  | 53 | 11.18 | 12 | 10.17 | 0.92 (0.48-1.77) | 0.753 | 12 | 10.81 | 0.99 (0.52-1.90) | 0.911 |
|  | **AA+AG** |  | 472 | 99.58 | 118 | 100 | - |  | 111 | 100 | - |  |
|  |  | **A** | 893 | 94.20 | 224 | 94.92 | 1 |  | 210 | 94.6 | 1 |  |
|  |  | **G** | 55 | 5.80 | 12 | 5.08 | 0.90 (0.48-1.68) | 0.670 | 12 | 5.4 | 0.96 (0.51-1.80) | 0.819 |
| rs9288953 | **CC** |  | 188 | 40.00 | 39 | 33.91 | 1 | 0.346 | 40 | 36.36 | 1 | 0.691 |
|  | **CT** |  | 218 | 46.38 | 62 | 53.91 | 1.37 (0.88-2.13) |  | 56 | 50.9 | 1.20 (0.77-1.88) |  |
|  | **TT** |  | 64 | 13.62 | 14 | 12.17 | 1.07 (0.55-2.09) |  | 14 | 12.73 | 1.05 (0.54-2.03) |  |
|  | **CT+TT** |  | 282 | 60.00 | 76 | 66.09 | 1.29 (0.84-1.98) | 0.230 | 70 | 63.64 | 1.16 (0.76-1.78) | 0.483 |
|  | **CC+CT** |  | 406 | 86.38 | 101 | 87.83 | 1.11 (0.60-2.04) | 0.684 | 96 | 87.27 | 1.06 (0.57-1.95) | 0.806 |
|  |  | **C** | 594 | 63.19 | 140 | 60.87 | 1 |  | 136 | 61.82 | 1 |  |
|  |  | **T** | 346 | 36.81 | 90 | 39.13 | 1.11 (0.82-1.49) | 0.514 | 84 | 38.18 | 1.06 (0.79-1.44) | 0.704 |
| rs1844089 | **GG** |  | 395 | 83.33 | 97 | 84.35 | 1 | 0.779 | 93 | 83.78 | 1 | 0.797 |
|  | **AG** |  | 77 | 16.24 | 17 | 14.78 | 0.92 (0.52-1.61) |  | 17 | 15.32 | 0.95 (0.54-1.68) |  |
|  | **AA** |  | 2 | 0.42 | 1 | 0.87 | - |  | 1 | 0.90 | - |  |
|  | **AG+AA** |  | 79 | 16.67 | 18 | 15.65 | 0.94 (0.54-1.64) | 0.793 | 18 | 16.22 | 0.91 (0.98-1.71) | 0.909 |
|  | **AG+GG** |  | 472 | 99.58 | 114 | 99,13 | - |  | 110 | 99.1 | - |  |
|  |  | **G** | 867 | 91.46 | 211 | 91.74 | 1 |  | 203 | 91.44 | 1 |  |
|  |  | **A** | 81 | 8.54 | 19 | 8.26 | 0.98 (0.59-1.64) | 0.890 | 19 | 8.56 | 1.02 (0.61-1.71) | 0.995 |
| rs11921669 | **CC** |  | 298 | 96.13 | 112 | 94.92 | 1 | 0.263 | 102 | 93.58 | 1 | 0.183 |
|  | **CT** |  | 12 | 3.87 | 5 | 4.24 | 1.17 (0.42-3.26) |  | 6 | 5.5 | 1.51 (0.57-4.01) |  |
|  | **TT** |  | 0 | 0 | 1 | 0.85 | - |  | 1 | 0.92 | - |  |
|  | **CT+TT** |  | 12 | 3.87 | 6 | 5.08 | 1.38 (0.52-3.65) | 0.577 | 7 | 6.42 | 1.75 (0.69-4.44) | 0.271 |
|  | **CC+CT** |  | 310 | 100.0 | 117 | 99.15 | - |  | 108 | 99.08 | - |  |
|  |  | **C** | 608 | 98.06 | 229 | 97.03 | 1 |  | 210 | 96.33 | 1 |  |
|  |  | **T** | 12 | 1.94 | 7 | 2.97 | 1.59 (0.64-3.98) | 0.361 | 8 | 3.67 | 1.96 (0.81-4.76) | 0.150 |
| rs2633582 | **AA** |  | 265 | 85.76 | 99 | 84.62 | 1 | 0.265 | 93 | 83.78 | 1 | 0.236 |
|  | **AC** |  | 44 | 14.24 | 17 | 14.53 | 1.05 (0.58-1.91) |  | 17 | 15.32 | 1.12 (0.61-2.04) |  |
|  | **CC** |  | 0 | 0 | 1 | 0.85 | - |  | 1 | 0.9 | - |  |
|  | **AC+CC** |  | 44 | 14.24 | 18 | 15.38 | 1.11 (0.62-2.00) | 0.765 | 18 | 16.22 | 1.18 (0.65-2.13) | 0.615 |
|  | **AA+AC** |  | 309 | 100 | 116 | 99.15 | - |  | 110 | 99.1 | - |  |
|  |  | **A** | 574 | 92.88 | 215 | 91.88 | 1 |  | 103 | 91.44 | 1 |  |
|  |  | **C** | 44 | 7.12 | 19 | 8.11 | 1.17 (0.67-2.03) | 0.619 | 19 | 8.56 | 1.24 (0.71-2.16) | 0.485 |

**Table 6** Distribution of genotypes and alleles frequencies of BTLA SNPs according to stage of the disease

|  |  |  |  | **Controls** | | **1** | **Stage** |  |  | **2** | **Stage** |  |  | **3 Stage** | | | | **4** | **Stage** |  |  |
| --- | --- | --- | --- | --- | --- | --- | --- | --- | --- | --- | --- | --- | --- | --- | --- | --- | --- | --- | --- | --- | --- |
| **SNP** |  | **Genotype** | **Allele** | **N** | **%** | **N** | **%** | **OR** | **p value** | **N** | **%** | **OR** | **p value** | **N** | **%** | **OR** | **p value** | **N** | **%** | **OR** | **p value** |
| rs2705511 |  | **AA** |  | 274 | 57.81 | 50 | 58.82 | 1 | 0.60 | 22 | 44.00 | 1 | 0.16 | 55 | 47.83 | 1 | **0.04** | 76 | 61 | 1 | 0.78 |
|  |  | **AC** |  | 170 | 35.86 | 32 | 37.65 | 1.04 |  | 23 | 46.00 | 1.68 |  | 46 | 40.00 | 1.35 |  | 41 | 33 | 0.87 |  |
|  |  | **CC** |  | 30 | 6.33 | 3 | 3.53 | 0.62 |  | 5 | 10.00 | 2.20 |  | 14 | 12.17 | 2.35 |  | 7 | 6 | 0.88 |  |
|  |  | **AC+CC** |  | 200 | 42.19 | 35 | 41.18 | 0.96 | 0.86 | 28 | 56.00 | 1.73 | 0.06 | 60 | 52.17 | 1.49 | **0.05** | 48 | 39 | 0.87 | 0.48 |
|  |  | **AA+AC** |  | 444 | 93.67 | 82 | 96.47 | 1.62 | 0.31 | 45 | 90.00 | 0.57 | 0.32 | 101 | 87.83 | 0.48 | **0.03** | 117 | 94 | 1.07 | 0.78 |
|  |  |  | **A** | 718 | 75.74 | 132 | 77.65 | 1 |  | 67 | 67.00 | 1 |  | 156 | 67.83 | 1 |  | 193 | 78 | 1 |  |
|  |  |  | **C** | 230 | 24.26 | 38 | 22.35 | 0.91 | 0.59 | 33 | 33.00 | 1.55 | 0.06 | 74 | 32.17 | 1.48 | **0.01** | 55 | 22 | 0.89 | 0.49 |
| rs1982809 |  | **AA** |  | 290 | 61.18 | 52 | 61.18 | 1 | 0.48 | 25 | 49.00 | 1 | 0.09 | 53 | 45.69 | 1 | **0.007** | 75 | 61 | 1 | 0.98 |
|  |  | **AG** |  | 159 | 33.54 | 31 | 36.47 | 1.09 |  | 20 | 39.00 | 1.46 |  | 52 | 44.83 | 1.79 |  | 41 | 33 | 1.00 |  |
|  |  | **GG** |  | 25 | 5.27 | 2 | 2.35 | 0.54 |  | 6 | 12.00 | 2.90 |  | 11 | 9.48 | 2.45 |  | 7 | 6 | 1.13 |  |
|  |  | **AG+GG** |  | 184 | 38.82 | 33 | 38.82 | 1.00 | 1.00 | 26 | 51.00 | 1.64 | 0.09 | 63 | 54.31 | 1.87 | **0.002** | 48 | 39 | 1.01 | 0.97 |
|  |  | **AA+AG** |  | 449 | 94.73 | 83 | 97.65 | 1.89 | 0.25 | 45 | 88.00 | 0.40 | 0.06 | 105 | 90.52 | 0.52 | 0.09 | 116 | 94 | 0.88 | 0.86 |
|  |  |  | **A** | 739 | 77.95 | 135 | 79.41 | 1 |  | 70 | 69.00 | 1 |  | 158 | 68.10 | 1 |  | 191 | 78 | 1 |  |
|  |  |  | **G** | 209 | 22.05 | 35 | 20.59 | 0.92 | 0.67 | 32 | 31.00 | 1.63 | **0.03** | 74 | 31.90 | 1.66 | **0.002** | 55 | 22 | 1.02 | 0.92 |
| rs9288952 |  | **AA** |  | 421 | 88.82 | 75 | 88.24 | 1 | 0.81 | 44 | 86.27 | 1 | 0.74 | 102 | 87.93 | 1 | 0.31 | 111 | 90 | 1 | 0.82 |
|  |  | **AG** |  | 51 | 10.76 | 10 | 11.76 | 1.14 |  | 7 | 13.73 | 1.38 |  | 12 | 10.34 | 1.00 |  | 12 | 10 | 0.92 |  |
|  |  | **GG** |  | 2 | 0.42 | 0 | 0 |  |  | 0 | 0 | 1.89 |  | 2 | 1.72 | 4.11 |  | 1 | 1 |  |  |
|  |  | **AG+GG** |  | 53 | 11.18 | 10 | 11.76 | 1.10 | 0.88 | 7 | 13.73 | 1.33 | 0.59 | 14 | 12.07 | 1.11 | 0.79 | 13 | 10 | 0.95 | 0.83 |
|  |  | **AA+AG** |  | 472 | 99.58 | 85 | 100 |  | 0.00 | 51 | 100 |  |  | 114 | 98.28 |  |  | 123 | 99 |  |  |
|  |  |  | **A** | 893 | 94.20 | 160 | 94.12 | 1 |  | 95 | 93.14 | 1 |  | 216 | 93.10 | 1 |  | 234 | 94 | 1 |  |
|  |  |  | **G** | 55 | 5.80 | 10 | 5.88 | 1.05 | 0.97 | 7 | 6.86 | 1.26 | 0.67 | 16 | 6.90 | 1.23 | 0.53 | 14 | 6 | 1.00 | 0.93 |
| rs9288953 |  | **CC** |  | 188 | 40.00 | 34 | 40.48 | 1 | 0.91 | 21 | 42.00 | 1 | 0.94 | 33 | 28.95 | 1 | **0.02** | 44 | 35 | 1 | 0.52 |
|  |  | **CT** |  | 218 | 46.38 | 40 | 47.62 | 1.01 |  | 23 | 46.00 | 0.94 |  | 70 | 61.40 | 1.82 |  | 59 | 48 | 1.15 |  |
|  |  | **TT** |  | 64 | 13.62 | 10 | 11.90 | 0.89 |  | 6 | 12 | 0.88 |  | 11 | 9.65 | 1.00 |  | 21 | 17 | 1.41 |  |
|  |  | **CT+TT** |  | 282 | 60.00 | 50 | 59.52 | 0.98 | 0.93 | 29 | 58.00 | 0.92 | 0.78 | 81 | 71.05 | 1.62 | **0.03** | 80 | 65 | 1.21 | 0.36 |
|  |  | **CC+CT** |  | 406 | 86.38 | 74 | 88.10 | 1.13 | 0.67 | 44 | 88.00 | 1.09 | 0.75 | 103 | 90.35 | 1.43 | 0.26 | 103 | 83 | 0.76 | 0.35 |
|  |  |  | **C** | 594 | 63.19 | 108 | 64.29 | 1 |  | 65 | 65.00 | 1 |  | 136 | 59.65 | 1 |  | 147 | 59 | 1 |  |
|  |  |  | **T** | 346 | 36.81 | 60 | 35.71 | 0.96 | 0.79 | 35 | 35.00 | 0.93 | 0.72 | 92 | 40.35 | 1.16 | 0.32 | 101 | 41 | 1.18 | 0.26 |
| rs1844089 |  | **GG** |  | 395 | 83.33 | 71 | 83.53 | 1 | 0.67 | 42 | 82.35 | 1 | 0.87 | 94 | 82.46 | 1 | 0.82 | 107 | 87 | 1 | 0.15 |
|  |  | **AG** |  | 77 | 16.24 | 13 | 15.29 | 0.96 |  | 9 | 17.65 | 1.14 |  | 19 | 16.67 | 1.05 |  | 14 | 11 | 0.69 |  |
|  |  | **AA** |  | 2 | 0.42 | 1 | 1.18 |  |  | 0 | 0 | 1.86 |  | 1 | 1.00 |  |  | 2 | 2 |  |  |
|  |  | **AG+AA** |  | 79 | 16.67 | 14 | 16.47 | 1.01 | 0.96 | 9 | 17.65 | 1.11 | 0.86 | 20 | 17.54 | 1.08 | 0.82 | 16 | 13 | 0.76 | 0.32 |
|  |  | **AG+GG** |  | 472 | 99.58 | 84 | 98.82 |  | 0.00 | 51 | 100 |  |  | 113 | 99.00 |  |  | 121 | 98 |  |  |
|  |  |  | **G** | 867 | 91.46 | 155 | 91.18 | 1 |  | 93 | 91.18 | 1 |  | 207 | 90.79 | 1 |  | 228 | 93 | 1 |  |
|  |  |  | **A** | 81 | 8.54 | 15 | 8.82 | 1.06 | 0.90 | 9 | 8.82 | 1.08 | 0.92 | 21 | 9.21 | 1.10 | 0.75 | 18 | 7 | 0.86 | 0.53 |

**Table 6** Distribution of genotypes and alleles frequencies of BTLA SNPs according to stage of the disease (continuation)

|  |  |  |  | **Controls** | | **1** | **Stage** |  |  | **2** | **Stage** |  |  | **3** | **Stage** |  |  | **4** | **Stage** |  |  |
| --- | --- | --- | --- | --- | --- | --- | --- | --- | --- | --- | --- | --- | --- | --- | --- | --- | --- | --- | --- | --- | --- |
| **SNP** |  | **Genotype** | **Allele** | **N** | **%** | **N** | **%** | **OR** | **p value** | **N** | **%** | **OR** | **p value** | **N** | **%** | **OR** | **p value** | **N** | **%** | **OR** | **p value** |
| rs11921669 |  | **CC** |  | 298 | 96.13 | 78 | 92.86 | 1 | 0.11 | 47 | 92.16 | 1 | - | 111 | 95.69 | 1 | - | 119 | 97 | 1 | 0.22 |
|  |  | **CT** |  | 12 | 3.87 | 5 | 5.95 | 1.67 |  | 4 | 7.84 | 2.26 |  | 5 | 4.31 | 1.18 |  | 3 | 2 | 0.70 |  |
|  |  | **TT** |  | 0 | 0 | 1 | 1.19 |  |  | 0 | 0 |  |  | 0 | 0 |  |  | 1 | 1 |  |  |
|  |  | **CT+TT** |  | 12 | 3.87 | 6 | 7.14 | 1.98 | 0.20 | 4 | 7.84 | 2.26 | 0.20 | 5 | 4.31 | 1.18 | 0.84 | 4 | 3 | 0.90 | 0.76 |
|  |  | **CC+CT** |  | 310 | 100 | 83 | 98.81 |  |  | 51 | 100 |  |  | 116 | 100 |  |  | 122 | 99 |  |  |
|  |  |  | **C** | 608 | 98.06 | 161 | 95.83 | 1 |  | 98 | 96.08 | 1 |  | 227 | 97.84 | 1 |  | 241 | 98 | 1 |  |
|  |  |  | **T** | 12 | 1.94 | 7 | 4.17 | 2.26 | 0.09 | 4 | 3.92 | 2.22 | 0.21 | 5 | 2.16 | 1.18 | 0.84 | 5 | 2 | 1.11 | 0.93 |
| rs2633582 |  | **AA** |  | 265 | 85.76 | 71 | 83.53 | 1 | 0.16 | 41 | 82.00 | 1 | - | 96 | 82.76 | 1 | 0.22 | 108 | 87 | 1 | 0.06 |
|  |  | **AC** |  | 44 | 14.24 | 13 | 15.29 | 1.13 |  | 9 | 18.00 | 1.37 |  | 19 | 16.38 | 1.21 |  | 14 | 11 | 0.80 |  |
|  |  | **CC** |  | 0 | 0 | 1 | 1.18 |  |  | 0 | 0 |  |  | 1 | 0.86 |  |  | 2 | 2 |  |  |
|  |  | **AC+CC** |  | 44 | 14.24 | 14 | 16.47 | 1.21 | 0.61 | 9 | 18.00 | 1.37 | 0.49 | 20 | 17.24 | 1.27 | 0.44 | 16 | 13 | 0.91 | 0.72 |
|  |  | **AA+AC** |  | 309 | 100 | 84 | 98.82 |  |  | 50 | 100 |  |  | 115 | 99.14 |  |  | 122 | 98 |  |  |
|  |  |  | **A** | 574 | 92.88 | 155 | 91.18 | 1 |  | 91 | 91.00 | 1 |  | 211 | 90.95 | 1 |  | 230 | 93 | 1 |  |
|  |  |  | **C** | 44 | 7.12 | 15 | 8.82 | 1.29 | 0.46 | 9 | 9.00 | 1.34 | 0.50 | 21 | 9.05 | 1.31 | 0.35 | 18 | 7 | 1.04 | 0.94 |

**Table. 7** Effect of variables on overall survival (Cox proportional hazards regression, univariate model) for all NSCLC patients, smoking NSCLC patients and never-smoking NSCLC patients

| **Kolumna1** | | All NSCLC patients | | | | Never-smoking NSCLC patients | | | | Smoking NSCLC patients | | | |
| --- | --- | --- | --- | --- | --- | --- | --- | --- | --- | --- | --- | --- | --- |
|  | | **Coefficient** | **p value** | **HR** | **95% CI** | **Coefficient** | **p value** | **HR** | **95% CI** | **Coefficient** | **p value** | **HR** | **95% CI** |
| Age | | 0.01 | 0.322 | 1.01 | 0.99-1.02 | 0.02 | 0.378 | 1.02 | 0.98 | 0.01 | 0.551 | 1.01 | 0.99-1.02 |
| Pack-years  continuous | | **0.02** | **0.000** | **1.02** | **1.01-1.03** | - | - | - |  | **0.03** | **0.000** | **1.03** | **1.02-1.04** |
| rs2705511 | A A | **0.13** | **0.046** | **1.29** | **1.00-1.65** | -0.22 | 0.262 | 0.30 | 0.30-1.38 | **0.19** | **0.007** | **1.45** | **1.11-1.91** |
| rs1982809 | A A | 0.12 | 0.055 | 1.27 | 0.99-1.63 | -0.08 | 0.668 | 0.40 | 0.40-1.81 | **0.16** | **0.019** | **1.38** | **1.06-1.81** |
| rs9288952 | A A | -0.17 | 0.070 | 0.71 | 0.48-1.03 | -0.37 | 0.144 | 0.18 | 0.18-1.29 | -0.13 | 0.209 | 0.77 | 0.50-1.16 |
| rs9288953 | C C | 0.03 | 0.767 | 1.17 | 0.77-1.78 | 0.11 | 0.740 | 0.42 | 0.42-6.18 | 0.03 | 0.752 | 1.13 | 0.72-1.77 |
|  | C T | 0.10 | 0.285 | 1.25 | 0.84-1.87 | 0.26 | 0.358 | 0.54 | 0.54-6.48 | 0.05 | 0.592 | 1.15 | 0.74-1.78 |
| rs1844089 | G G | -0.07 | 0.391 | 0.86 | 0.62-1.21 | -0.37 | 0.144 | 0.18 | 0.18-1.29 | -0.02 | 0.804 | 0.95 | 0.66-1.38 |
| rs11921669 | C C | 0.26 | 0.109 | 1.68 | 0.89-3.16 | 0.00 |  |  |  | 0.30 | 0.077 | 1.82 | 0.94-3.56 |
| rs2633582 | A A | -0.07 | 0.411 | 0.87 | 0.62-1.22 | -0.37 | 0.144 | 0.48 | 0.18-1.29 | -0.02 | 0.830 | 0.96 | 0.66-1.39 |
| gender | M | **0.20** | **0.006** | **1.48** | **1.12-1.96** | 0.16 | 0.426 | 1.37 | 0.63-3.00 | **0.17** | **0.039** | **1.40** | **1.02-1.92** |
| smoking | tak | 0.15 | 0.130 | 1.36 | 0.91-2.04 | - | - | - | - | - | - | - | - |
| Pack-years  categories | 0-10 | -0.54 | 0.597 | 0.58 | 0.08-4.30 | - | - | - | - | - | - | - | - |
|  | 11 - 20 | 0.07 | 0.772 | 1.08 | 0.65-1.77 | - | - | - | - | - | - | - | - |
|  | 21 - 30 | -0.05 | 0.839 | 0.95 | 0.59-1.52 | - | - | - | - | - | - | - | - |
|  | 31 - 40 | **0.45** | **0.045** | **1.58** | **1.01-2.47** | - | - | - | - | - | - | - | - |
|  | >40 | **0.79** | **<0.001** | **2.20** | **1.39-3.36** | - | - | - | - | - | - | - | - |
| NSCLC histological type | AD | 0.13 | 0.477 | 1.18 | 0.47-2.97 | - | - | - | - | - | - | - | - |
|  | LCC | -0.10 | 0.698 | 0.81 | 0.45-1.46 | - | - | - | - | - | - | - | - |
|  | SCC | -0.15 | 0.738 | 0.77 | 0.24-2.44 | - | - | - | - | - | - | - | - |
| Clinical Stage | II | **0.47** | **0.041** | **1.60** | **1.02-2.53** | -0.13 | 0.983 | <0.01 | 0-NA | 0.39 | 0.101 | 1.49 | 0.93-2.39 |
|  | III | **1.03** | **<0.001** | **2.80** | **1.94-4.05** | **2.14** | **0.002** | **8.51** | **2.25-31.12** | **0.88** | **0.000** | **2.42** | **1.62-3.61** |
|  | IV | **2.13** | **<0.001** | **8.38** | **5.70-12.30** | **1.22** | **0.000** | **14.27** | **3.35-60.72** | **2.08** | **0.000** | **7.98** | **5.28-12.05** |
| Surgery | yes | **-0.89** | **0.000** | **0.17** | **0.13-0.23** | **-0.92** | **0.000** | **0.16** | **0.07-0.38** | **-0.89** | **0.000** | **0.17** | **0.12-0.23** |
| HR - hazard ratio; 95% CI – confidence interval | | | | | | | | | | | | | |


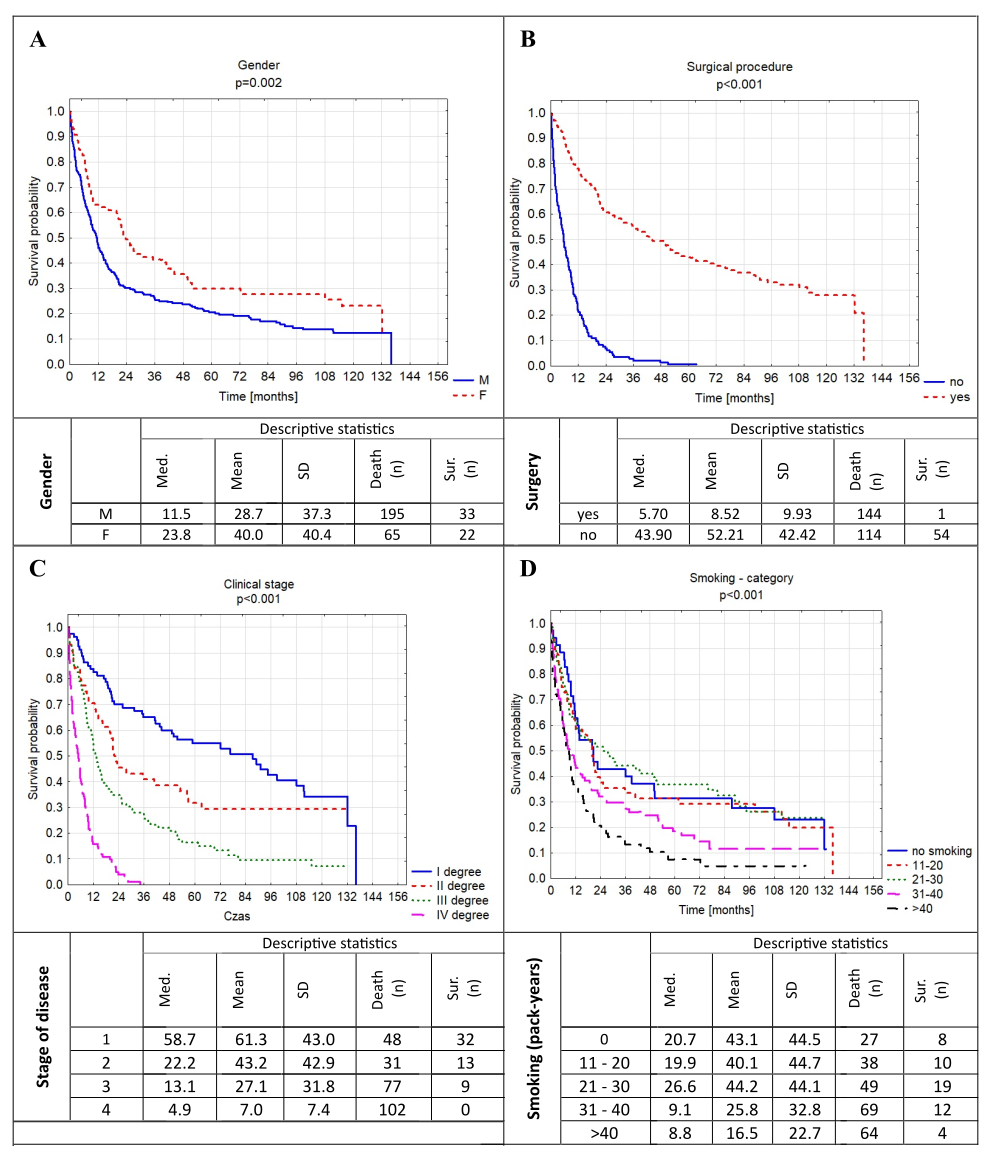


**Fig. 1** Probability of survival in relation to clinical factors: **A**) gender; **B**) surgery or no; **C**) stage of disease; **D**) category – pack-years. Med – median; SD – standard deviation; sur. – survival; n – number of patients.


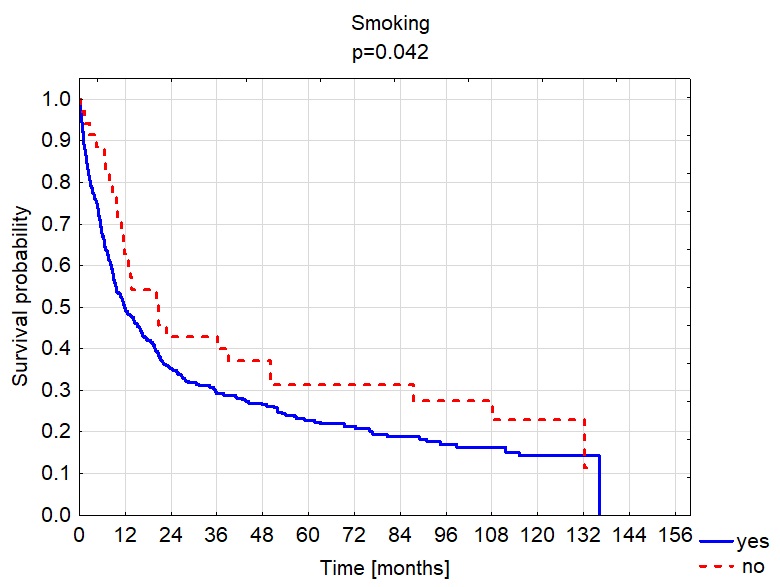


|  | | Descriptive statistics | | | | |
| --- | --- | --- | --- | --- | --- | --- |
|  |  | Median | Mean | SD | Death (n) | Survival (n) |
| Smoking | yes | 11.9 | 30.8 | 37.8 | 221 | 46 |
|  | no | 20.7 | 43.1 | 44.5 | 27 | 8 |

**Fig. 2** Probability of survival in relation to smoking status in NSCLC patients. SD – standard deviation; n – number of patients.
